# Large Differences in Herbivore Performance Emerge From Simple Herbivore Behaviours and Fine‐Scale Spatial Heterogeneity in Phytochemistry

**DOI:** 10.1111/ele.70044

**Published:** 2024-12-31

**Authors:** Vincent S. Pan, Enakshi Ghosh, Paul J. Ode, William C. Wetzel, Kadeem J. Gilbert, Ian S. Pearse

**Affiliations:** ^1^ Department of Integrative Biology Michigan State University East Lansing Michigan USA; ^2^ W. K. Kellogg Biological Station Michigan State University Hickory Corners Michigan USA; ^3^ Ecology, Evolution, and Behavior Program Michigan State University Easting Lansing Michigan USA; ^4^ Department of Agricultural Biology Colorado State University Fort Collins Colorado USA; ^5^ Graduate Degree Program in Ecology Colorado State University Fort Collins Colorado USA; ^6^ Land Resources and Environmental Sciences Montana State University Bozeman Montana USA; ^7^ Department of Plant Biology Michigan State University East Lansing Michigan USA; ^8^ U.S. Geological Survey, Fort Collins Science Center Fort Collins Colorado USA

**Keywords:** 1/f noise, arrestment, herbivore foraging, insect movement, integrated step selection analysis, phytochemistry, plant defence, spatial autocorrelation, spatial heterogeneity, trait variation

## Abstract

Patterns of phytochemistry localisation in plant tissues are diverse within and across leaves. These spatial heterogeneities are important to the fitness of herbivores, but their effects on herbivore foraging and dietary experience remain elusive. We manipulated the spatial variance and clusteredness of a plant toxin in a synthetic diet landscape on which individual caterpillars fed. We monitored caterpillars with cameras across most of their larval development. Caterpillars that fed on diets with a lower spatial variance and more clustered arrangement of toxins had overall worse performance, mostly because those caterpillars ate less, moved more, ingested more toxin, or failed to physiologically acclimate. Using empirically parameterised individual‐based models, we found that differences in movement away from, not towards, less toxic food drove a body size‐dependent effect of clusteredness. Hence, the spatial pattern of phytochemicals itself, beyond mean concentration, can have important consequences for herbivores through complex interactions with herbivore foraging.

## Graphical abstract

Patterns of phytochemistry localisation in plant tissues are diverse within and across leaves. Simple foraging behaviours of insect herbivores can have nontrivial emergent interactions with fine‐scale heterogeneity in phytochemistry, a scale relevant to the foraging behaviour of herbivores, but which is often overlooked in studies of phytochemistry. Both spatial variance and arrangement of phytochemistry affected the performance and space use of herbivores and did so at a magnitude that rivalled the effects of mean phytochemical concentration.

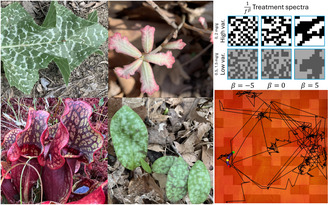

## 1 Introduction

**FIGURE 1:**
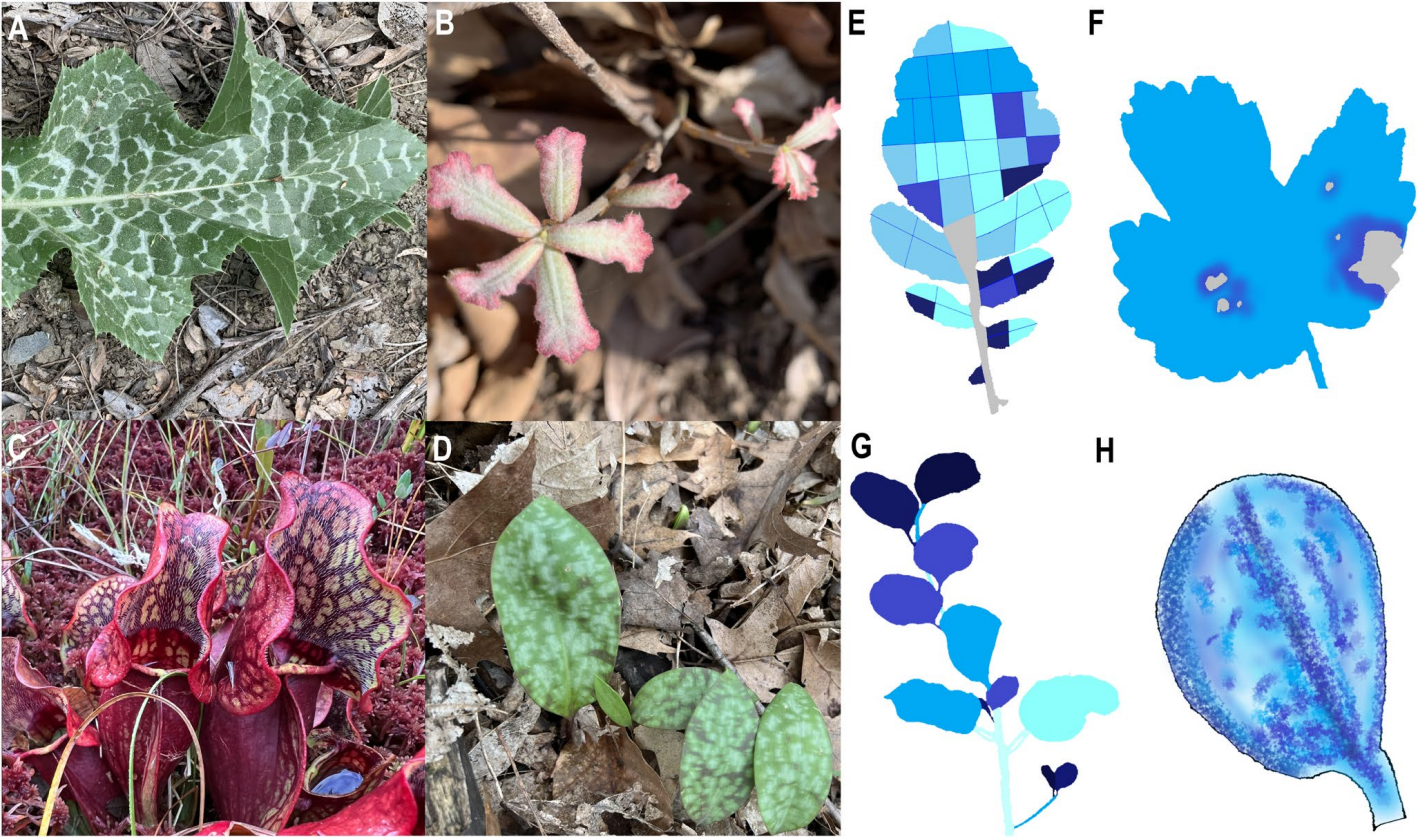
(A–D) Examples of fine‐scale spatial variation in plant quality within leaves are ubiquitous in nature, sometimes visually apparent with pigmentation differences. (E–H) Redrawing of figures from studies that specifically measured the spatial variation in defensive compounds. Darker colours represent greater toxin concentrations. Grey represents missing data. (E) Glucosinolate variation in a *Raphanus sativus* leaf (Shelton 2005). (F) Furanocoumarin variation in *Pastinaca sativa* after leaf damage (Zangerl et al. 2002). (G) Cyanide variation in a *Eucalyptus cladocalyx* branch (Gleadow and Woodrow 2000). (H) Glucosinolate variation in an *Arabidopsis thaliana* leaf (Shroff et al. 2008). The photographed species by VSP are (A) *Silybum marianum* , (B) *Quercus marilandica* , (C) *Sarracenia purpurea* and (D) *Erythronium americanum* .

Animals live in a mosaic environment in which resources, predator risk and abiotic conditions each vary across spatial scales. At a fine scale, variation in habitat quality drives the distribution of animals (Zucker 1982), their fitness (Schultz 1983b) and their ability to cope with unfavourable large‐scale environmental conditions (Woods, Dillon, and Pincebourde 2015). Insect herbivores face micro‐scale environmental heterogeneity at the plant organ and sub‐organ level (< 20 cm, Pincebourde and Woods 2020) in which phytochemistry can exhibit tremendous variation (Figure 1, Orians, Pomerleau, and Ricco 2000). For instance, at a gain of ~1 cm, toxins vary by 10–100‐fold within single leaves of both *Arabidopsis thaliana* and *Raphanus raphanistrum* (Shelton 2005; Shroff et al. 2008). There is increasing interest in how herbivorous insects interact with spatial variation in plant quality, especially at the scale of individual plants or plant communities (Barbosa et al. 2009; Hauri, Glassmire, and Wetzel 2021; Hauri et al. 2022; Salazar and Marquis 2022), but little is known about the effects of fine‐scale phytochemical heterogeneity on the experience and performance of individual herbivores. Understanding phytochemical heterogeneity at this scale may be important. Studies generally explain the effect of phytochemistry from information on the whole plant, but at a finer scale, an individual herbivore's experience of phytochemistry may be decoupled from that of the whole plant (Potter, Arthur Woods, and Pincebourde 2013). Likewise, important emergent effects of heterogeneity can occur that cannot be captured by standard experimental designs, such as no‐choice and choice assays, aimed only at measuring the effect of mean phytochemistry level.

One such emergent effect of heterogeneity is that variation per se can suppress herbivore performance (Wetzel et al. 2016, 2023). Thus, organ and tissue scale variation in plant defence localisation itself has been proposed to act as a plant defence (Schultz 1983a, 1983b; Whitham 1981). A negative effect of phytochemical variation on herbivore fitness may be expected because many herbivore performance functions are concave; accordingly, variation reduces herbivore performance via nonlinear averaging (Herrera 2009; Karban, Agrawal, and Mangel 1997). More recently, this argument was extended to include physiological dynamics (Pearse, Paul, and Ode 2018; Wetzel and Thaler 2016), wherein the physiological cost and limitations to tracking chemical variation reduce herbivore performance. Others note that phytochemical variation forces herbivores to move and make foraging choices, with potentially positive or negative effects on herbivore fitness (Bernays 2001; Schultz 1983a, 1983b; Thiel et al. 2020, 2021). Each of these processes likely contributes to the net fitness effect of phytochemical variation to herbivores; however, two open questions remain.

First, how herbivores experience the spatial variation in phytochemistry available in the microenvironment is unclear. Despite the attractive suggestion that increased spatial variance in phytochemical concentration across an agricultural landscape can be harnessed to suppress herbivores (Wetzel et al. 2016; Pearse, Paul, and Ode 2018), experimental evidence is mostly from force‐feeding experiments that manipulated temporal variation in phytochemistry (Pearse, Paul, and Ode 2018). Time‐for‐space substitutions may be inappropriate because they involve different processes that may have contrasting effects (Perret, Evans, and Sax 2024), and individual processes may not be ergodic. While greater spatial variance can lead to greater temporal variance experienced by an individual, the extent is likely dependent on the foraging behaviour of the herbivore. However, much remains unknown about how insect herbivores make fine‐scale foraging decisions, and existing theories suggest that different herbivore behaviours can substantially alter the magnitude and direction of the effect of phytochemical variability on herbivore performance (Shelton 2000; Thiel et al. 2020, 2021). Indeed, many other models of plant‐herbivore interactions are sensitive to assumptions about the specific behaviour of herbivores, which are difficult to verify (Lewis 1994; Riolo, Rohani, and Hunter 2015; Root 1973; Underwood, Inouye, and Hambäck 2014). Recent advances in the modelling of large‐bodied animal foraging behaviour (Avgar et al. 2016) and animal tracking technologies (Nathan et al. 2022) provide a way to address this key gap empirically. Using high‐resolution data on the location and environment of individual herbivores, one can construct realistic individual‐based behavioural models. The effects of environmental heterogeneity can then be evaluated under different behavioural models and validated with data.

Second, when there is spatial variation in phytochemistry, how its specific arrangement affects herbivores is unclear (Wetzel and Whitehead 2020). ‘Clustered’, or autocorrelated, arrangements of variation are common in nature (Halley 1996; Vasseur and Yodzis 2004) and lead to a positive coupling between the amount of variance and spatial scale (Denny 2015). Clusteredness of plant defences within the possible range of herbivore movement is of particular interest because even subtle differences in herbivore behaviour will change the local landscape over which herbivores experience their environment (Sears and Angilletta 2015). The clusteredness in phytochemistry could increase the distance required to travel to different resources (Schultz 1983a; Shelton 2004). It could also increase the amount of information available to the herbivore about what is nearby (Schmidt, Massol, and Szymkowiak 2022) but decrease the amount of information available about the highest quality food available in a landscape, and thus lead to poor foraging choices. Nevertheless, despite the known importance of clusteredness to many ecological processes in theoretical studies (Cuddington and Yodzis 1999; Ruokolainen et al. 2009; Yang et al. 2019), few empirical experiments to date have considered it as a property of biological interest (Gajewski et al. 2024; Gonzalez and Holt 2002; Hauri et al. 2022), rather than a statistical nuisance (Legendre 1993).

**FIGURE 2:**
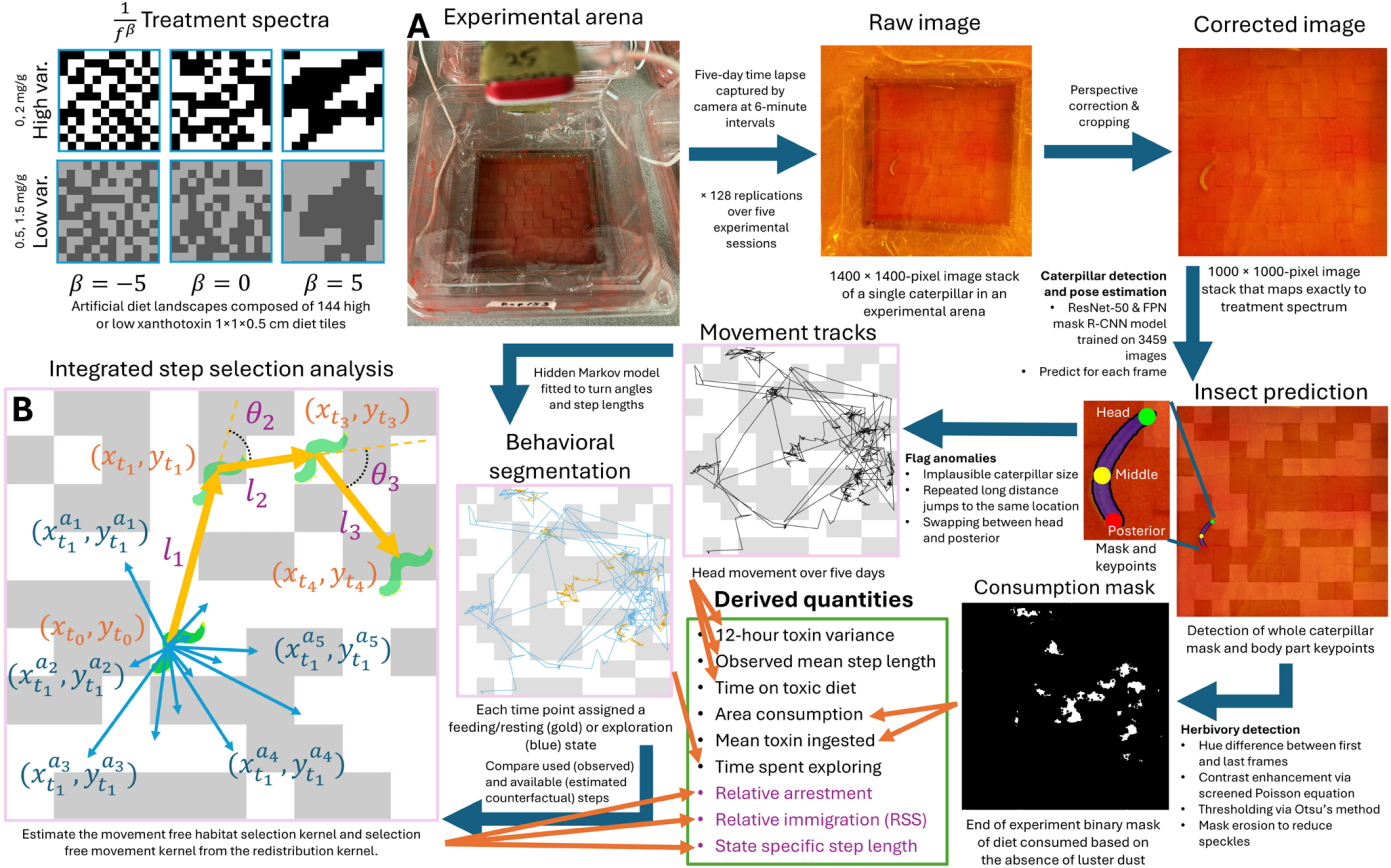
Summary of the data processing pipeline with which we derived measures of herbivore movement and feeding behaviour to explain herbivore performance. Lighter squares represent diet tiles that contain a higher concentration of xanthotoxins. (A) An example experimental arena monitored by a camera. A layer of red lustre dust was sprayed on top of the diet to delineate eaten and uneaten diet. (B) Illustration of the analysis of caterpillar movement over four discrete time points in an x,y coordinate space. The actual steps taken by the caterpillar are indicated by the yellow arrows, with l as step length and θ as turn angle. The available steps at $t_{0}$ are indicated by the blue arrows. Derived quantities in black were used in a structural equation model to explore possible proximal mechanisms that explain treatment effects. Derived quantities in purple were used to clarify the causal behavioural and movement mechanisms using an *in silico* experiment. More details can be found in Appendices S9–S12 and Video S1.

To address these gaps, we conducted a controlled experiment in which we manipulated the spatial patterns (variance and clusteredness) of the phytochemical xanthotoxin in artificial diets. Xanthotoxin is broadly toxic to many organisms by intercalating DNA strands, preventing transcription (Berenbaum 1978). We observed the foraging behaviour and performance of a caterpillar, *Trichoplusia ni* (Noctuidae), feeding in our artificial micro environment (Figure 2a,b). In nature, *T. ni* can sense the concentration of xanthotoxin and feeds on xanthotoxin‐containing Apiaceae and Rutaceae plants (Sutherland and Greene 1984), with concentrations on par with those used in our experiment. Notably, xanthotoxin displays substantial variation within and across organs, like many phytochemicals (Berenbaum and Zangerl 1986).

We tested the hypothesis that greater spatial variance and lower clusteredness (i.e. ‘dispersed’) enhance the performance of herbivores by facilitating foraging. With a more variable and more dispersed toxin distribution, we predict that herbivores would spend less time on more toxic diets and ingest lower levels of toxins. However, we predict that greater spatial variance and dispersion leads to lower consumption, greater movement and greater temporal variance experienced by the herbivores that dampen the benefit of selective feeding. Finally, we parameterised different individual‐based models to identify the minimum set of behavioural rules that can reproduce our observed treatment effects in an *in silico* experiment. We looked at our treatment effects on the scale of movement and two aspects of caterpillar foraging behaviour: (1) their tendency to reduce movement and spend longer time on preferred food and (2) their tendency to move towards preferred food. We use the term ‘arrestment’ for reductions in movement and the term ‘immigration’ for movements towards favoured diets (Avgar et al. 2016; Kennedy 1978). While both mechanisms lead to the identical observation that caterpillars spend more time on their favoured diet, a common measure of preference, our high‐resolution data allow us to disentangle the two. Taken together, our results show how spatial heterogeneity in phytochemistry, thought to be an important driver of plant‐herbivore interactions in the field, can affect herbivore performance through emergent interactions with simple fine‐scale behaviours.

## 2 Methods

### 2.1 Caterpillar Performance on an Artificial Landscape

To test how different degrees of spatial clusteredness and spatial variance in toxin concentration affect herbivore performance, we constructed 128 artificial diet landscapes (appendices 1–2, Figure 2b), each enclosing a single *T. ni* caterpillar, reared from eggs on xanthotoxin‐free artificial diet. We assembled each 12 × 12 cm diet landscape from smaller 1 × 1 × 0.5 cm artificial diet tiles into which we incorporated either a high‐ or low‐concentration of xanthotoxin (8‐methoxypsoralen). Our experiment consisted of a full factorial cross between two variation treatments (high or low) and three clusteredness treatments (negatively autocorrelated, not correlated and positively autocorrelated) (Figure 2a). Both high and low variation treatments kept the mean toxin concentration at 1 mg/g but alternated the high and low concentration between either 0 and 2 mg/g or 0.5 and 1.5 mg/g. For different clusteredness treatments, we arranged the diet tiles in patterns generated by a spectral synthesis method (Appendix S3) in which we simulated noise belonging to the $\frac{1}{f^{\beta }}$ inverse power law family. This family of noise is common in nature (Vasseur and Yodzis 2004) and arises naturally from many types of stochastic processes (Gisiger 2001; Kendal and Jørgensen 2011). They hold that the variance of a frequency f scales as $\frac{1}{f^{\beta }}$, where β is the spectral exponent. When β>0, long‐range frequencies dominate; when β<0, short‐range frequencies dominate; when β=0, all frequencies have equal variance. We used β = −5, 0, and 5 for the negatively autocorrelated (dispersed), not correlated (spatially random) and positively autocorrelated (clustered) treatments, respectively (Figure 2). Given that the noise follows a power law, beyond the grain size of 1 cm^2^ the treatments are dispersed, random or clustered at all scales.

To test how herbivores perform on different diet landscapes, we placed an individual *T. ni* larva on each diet landscape. We used caterpillars ranging from early second to late fourth instar to test whether treatment effects were consistent across caterpillar developmental stages (with different movement and sensory capacities, Wang et al. 2023). Each caterpillar was weighed before ($m_{0}$) and after the experiment ($m_{t}$), which lasts for five days. Some caterpillars that started to spin silk during the experiment were weighed immediately upon onset of the behaviour. From these data, we calculated the hourly relative growth rate (RGR) when the caterpillar was exposed to the diet averaged over the period of t as $\frac{\ln m_{0}-\ln m_{t}}{t}$. We allowed the caterpillars to feed on the same diet after the experiment until they pupated. We recorded pupation, adult eclosure, and survival status every 1–2 days. Thus, we had a total of four different caterpillar performance metrics for downstream analyses: RGR, time to pupation, eclosure probability and survival. We conducted a total of 128 trials (*n* = 20–23 / treatment) across five sessions over 5 weeks.

### 2.2 Caterpillar Performance Analysis

To test how landscape spatial variance and arrangement, and caterpillar size, interactively affect caterpillar performance, we fitted caterpillar RGR (*n* = 111), time to pupation (*n* = 96) and eclosure probability (*n* = 128) in individual generalised linear mixed models (package *glmmTMB* ver. 1.1.7, Brooks et al. 2017). Caterpillar survival (*n* = 128), which was right censored at the time of pupation if the insect never eclosed, was fitted in a Cox‐proportional hazard model (package *survival* ver. 3.3–1, Therneau 2020). We used an identity‐linked normal distribution, a log‐linked gamma distribution and a logit‐linked binomial distribution for the conditional distribution of RGR, time to pupation and eclosure respectively. In each model, we included variation treatment, clusteredness treatment, log caterpillar pre‐weight and the interaction between log pre‐weight and both treatments as fixed effects. All models except for eclosure exhibited strong nonlinearity in the residuals, so we also added a quadratic log pre‐weight term. Experimental session identity was added as a random intercept in the mixed models or as a conditional variable in the survival analysis, although it explained very little variance across all models.

### 2.3 Estimating Proximal Variables That Explain Performance

To test how herbivore movement and feeding behaviour influenced performance, we set up individual cameras that photographed each arena every 6 min over 5 days. From the camera data, we measured five proximal variables that might explain differences in RGR. We derived (1) average variance in toxin concentration exposure over an arbitrarily chosen 12 h sliding window (refer to Fang et al. 2007, who showed that cytochrome P450 expression peaked 12 h after exposure to the insecticide fenvalerate in *T. ni* ) and (2) observed log mean step length (Euclidean displacement over six minutes) from the raw movement tracts. We derived (3) the log area of the artificial diet consumed and (4) the mean concentration of toxin ingested from the final binary mask of the eaten diet. We derived (5) the proportion of time spent exploring from movement tracks labelled by individually fitted Hidden Markov Models. We also measured the proportion of time spent on the more toxic diet to explain variation in these five proximal variables. The steps of the data processing pipeline that arrive at these quantities are summarised in Figure 2 and detailed in appendices 3–8.

### 2.4 Structural Equation Model

**FIGURE 3:**
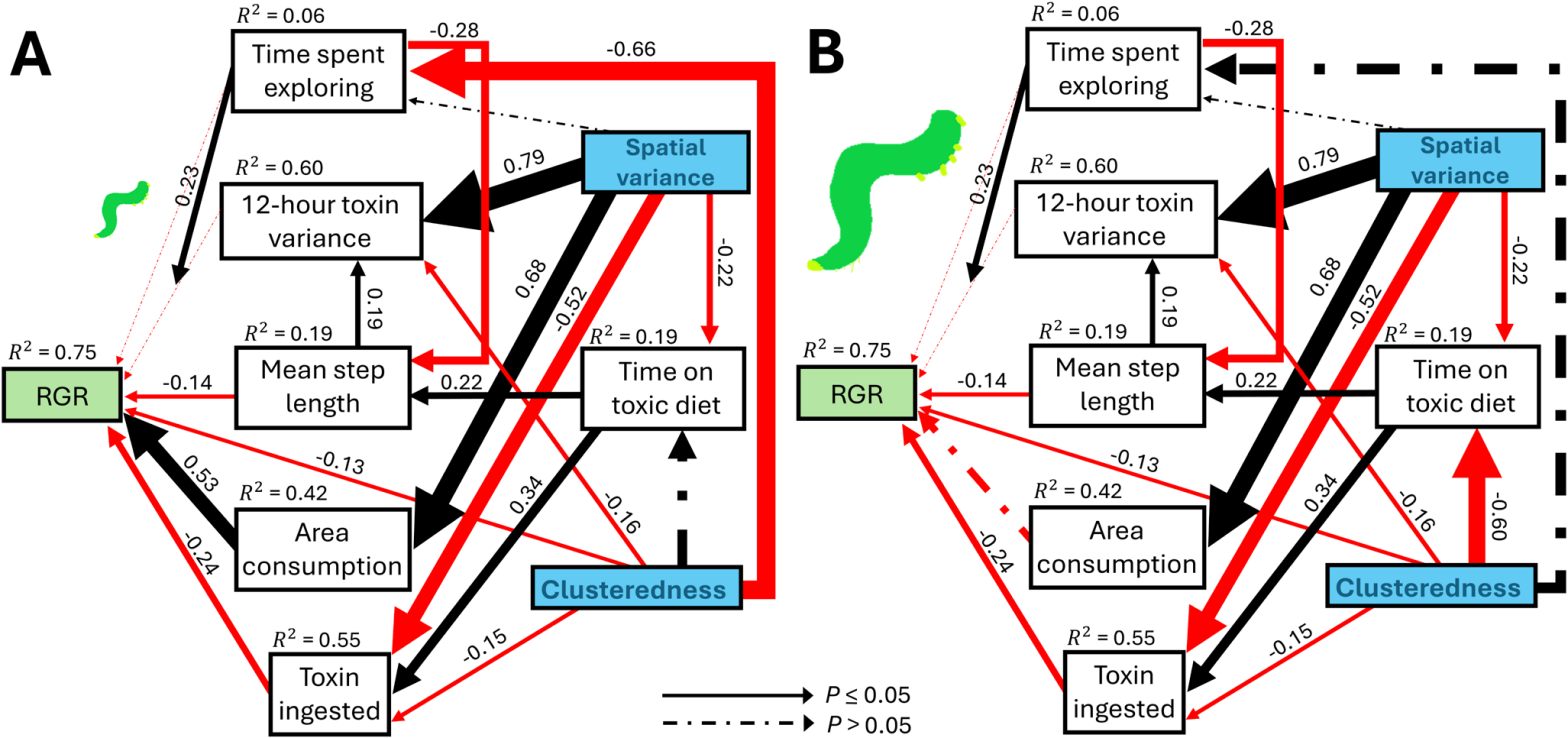
Structural equation model of proximal mechanisms by which experimental treatments (blue bolded text) may have affected relative growth rate (RGR) of small (A) or large (B) caterpillars. For clarity, clusteredness (β) is modelled as a continuous variable and we also omitted caterpillar size from the graph. Though we modelled caterpillar sizes as a continuous variable, we display the conditional estimates of caterpillars with size ±2 standard deviations in the experiment (small and large). Only the standardised path coefficients of significant paths are displayed. Red and black arrows indicate a negative and positive causal effect, respectively. The size of the arrows was scaled by the magnitude of the path coefficient. Path coefficients were standardised by the observed standard deviation of continuous response and predictor variables. Marginal $R^{2}$ of each sub‐model is displayed next to the response variable. *d‐*separation test (Shipley 2009), package *piecewiseSEM* ver. 2.3.0 (Lefcheck 2016) revealed no significant missing paths (*Fisher's C* = 42, *df* = 42, *p* = 0.45). Mean proportion of time spent on toxic diets and variance in toxin concentration experienced have no obvious directed causal relationship but exhibit correlated errors because the mean–variance relationship of the beta distribution is constrained.

To explore the relative strengths of different causal mechanisms on herbivore performance, we performed a confirmatory piecewise path analysis on RGR, our five proximal variables, time on toxic diet and three treatment variables. We constructed seven sub‐models using a subset of the data with complete observations (*n* = 87). Proportions of time on a toxic diet and proportions of time exploring were logit transformed (Warton and Hui 2011). All variables were modelled with an identity‐linked normal distribution. For simplicity, we modelled clusteredness as a continuous variable, although the results do not differ from when we modelled it as a discrete variable as before. Our explicit structural hypotheses can be found in Figure 3. We added session identity as a random intercept in all models.

### 2.5 Estimation and Simulation With Different Behavioural Rules

To identify the salient behavioural mechanisms that explain the treatment effect of clusteredness, we performed integrated Step Selection Analysis (iSSA) to estimate behavioural parameters for constructing different individual‐based models that include various combinations of behavioural rules. We first conducted an exploratory analysis to investigate if there exist systematic differences in behavioural parameters among treatments and different sizes of caterpillars. With the tentative set of explanatory behavioural differences, we performed an *in silico* experiment in which we simulated different behavioural models under different clusteredness regimes. Doing so allows us to identify the minimum required details of behaviour that can reproduce the effects of clusteredness and disentangle causality between movement, behavioural and physiological state variables that in reality exhibit complicated dynamic feedback. Accordingly, we considered changing three behaviours, including (i) relative arrestment on the less toxic diet relative to the more toxic diet, (ii) relative immigration onto a less toxic diet relative to a more toxic diet (see Kennedy (1978) for further discussion) and (iii) different scales of movement. More details are reported in appendices 9–12.

Deep learning was conducted in Python ver. 3.8.18 (Van Rossum and Drake 2009) with other computer vision tasks done with opencv (package *cv2* ver. 4.8.1, Bradski 2000, appendices 4–7). All other data processing and analyses were conducted in R ver. 4.3.1 (R Core Team 2023) and C++ using the g++ compiler ver. 12.2.0 (Free Software Foundation Inc. 2022). Image processing in R was done with the CImg library interface *imager* ver. 0.42.13 (Barthelme et al. 2024). We evaluated the significance of regression terms using Wald $\chi^{2}$ tests. Non‐significant interactions were dropped, and the model refitted. Post hoc contrasts between treatment groups were performed using the Tukey method (package *emmeans* ver. 1.7.5, Lenth et al. 2024). For contrasts that involve an interaction with pre‐weight, we used ±2 standard deviations as a stand‐in for small and large caterpillars. All reported mean differences were standardised by the standard deviation to aid interpretation.

## 3 Results

### 3.1 Treatment Effects on Caterpillar Performance

**FIGURE 4:**
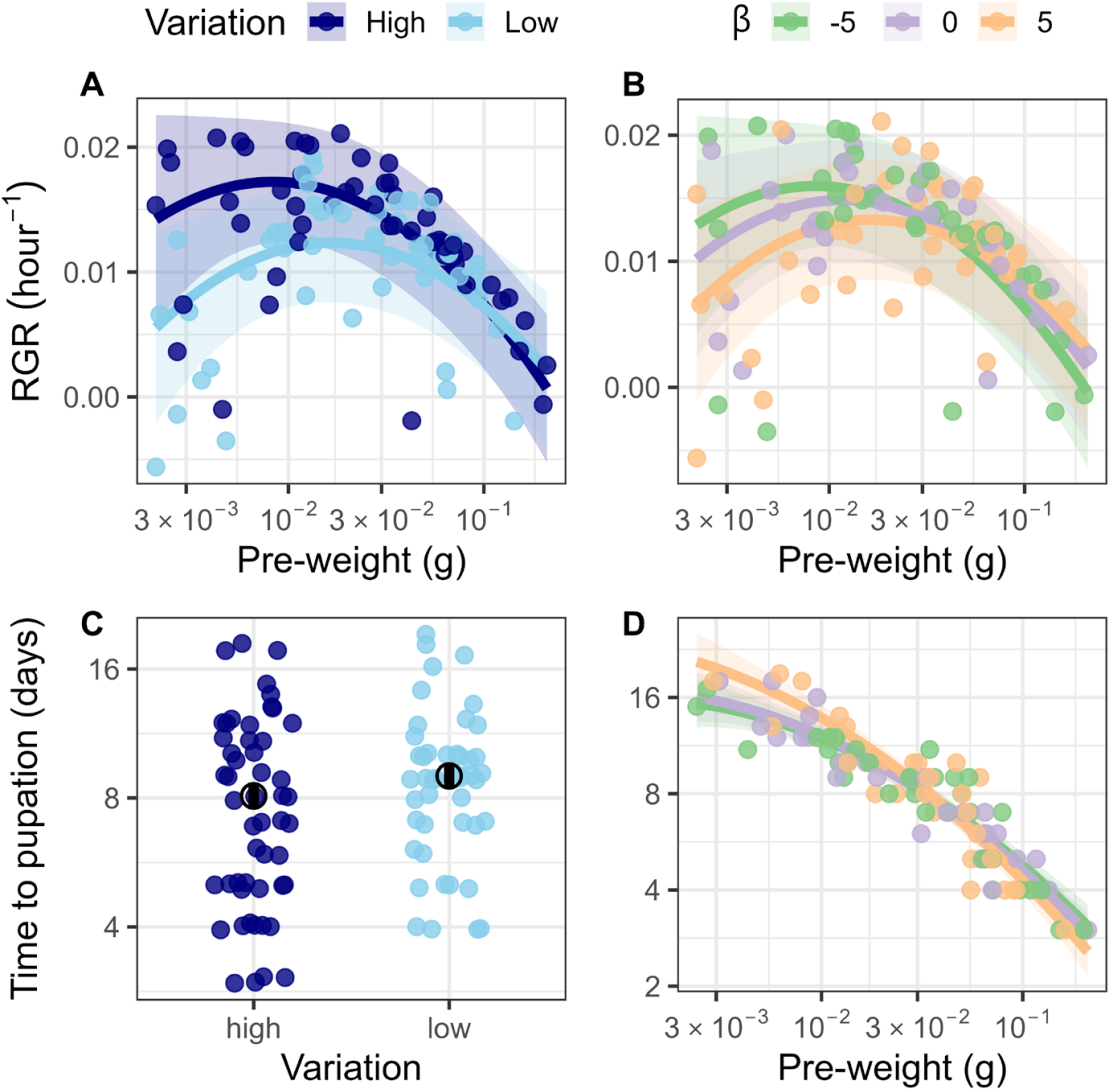
(A, B) Relative growth rate (RGR) (marginal $R^{2}$ = 0.44) was higher and (C, D) time to pupation (marginal $R^{2}$ = 0.88) was shorter for small caterpillars in either the high variation treatment or the more spatially dispersed treatments (smaller β). These effects attenuated (A, B) or slightly reversed (D) for larger caterpillars. Each point represents a caterpillar. Ribbons and error bars show 95% confidence intervals. Solid lines and open circles show mean estimates.

Caterpillars that fed on a more variable diet had an overall faster growth rate and shorter development time, but the effect on RGR was most pronounced for small caterpillars (Figure 4A, size × variation: $\chi^{2}$(1) = 10.7, *p* = 0.0011). An additional experiment with no variation (constant 1 mg/g) as a treatment produced the same pattern, in which caterpillar performance was lower on a no‐variation diet (Appendix S13, Figure S1). Small caterpillars had a 1.5 standard deviation (SD) (mean [95% confidence intervals]; [0.88, 2.1] SD, *t* = 4.8, *p* < 0.001) higher RGR in the high variation treatment, but for large caterpillars, RGR did not differ between variation treatments (−0.29 [−0.86, 0.29] SD, *t* = −0.99, *p* = 0.33). The high variation treatment also caused a 10% ([−16, −4.2] %, $\chi^{2}$(1) = 10.5, *p* = 0.0012) decrease in time to pupation regardless of initial body size (Figure 4C, size × variation: $\chi^{2}$(1) = 0.47, *p* = 0.50). There was no effect of high variation treatment on eclosure probability ($\chi^{2}$(1) = 0.0002, *p* = 0.99) or survival ($\chi^{2}$(1) = 0.12, *p* = 0.73), and the effects were not dependent on caterpillar size (eclosure_size × variation_: $\chi^{2}$(1) = 0.23, *p* = 0.63; survival_size × variation_: $\chi^{2}$(1) = 0.67, *p* = 0.41).

The effects of a more clustered diet arrangement generally decreased with caterpillar size and were strongest in the clustered treatment (RGR_size × clusteredness_: $\chi^{2}$(2) = 6.5, *p* = 0.039, Figure 4B; time to pupation_size × clusteredness_: $\chi^{2}$(2) = 7.7, *p* = 0.021, Figure 4D). For small caterpillars, the clustered treatment decreased RGR by a sizable 1.1 SD ([−1.9, −0.35] SD, *t* = −2.8, *p* = 0.016) and increased time to pupation by 37% ([9.5, 71] %, *t* = 2.8, *p* = 0.016) relative to the dispersed treatment. Caterpillars in the clustered treatment also had 30% longer time to pupation compared to the spatially random treatment ([4.3, 63] %, *t* = 2.3, *p* = 0.052). There was no difference in RGR between the clustered and spatially random (−0.62 [−1.3, 0.090] SD, *t* = −1.7, *p* = 0.21) and between the spatially random and dispersed treatments (−0.47 [−1.2, 0.28] SD, *t* = −1.2, *p* = 0.45), and in time to pupation between the spatially random and dispersed treatments (5.0 [−14, 26] %, *t* = 0.48, *p* = 0.88) for small caterpillars. For large caterpillars, there was no difference between any of the clusteredness treatments in RGR ($\beta_{5}$ vs. $\beta_{0}$: *t* = 0.72, *p* = 0.75; $\beta_{5}$ vs. $\beta_{-5}$: *t* = 1.7, *p* = 0.19; $\beta_{0}$ vs. $\beta_{-5}$: *t* = 1.0, *p* = 0.57), and in time to pupation ($\beta_{5}$ vs. $\beta_{0}$: *t* = −1.5, *p* = 0.29; $\beta_{0}$ vs. $\beta_{-5}$: *t* = −0.64, *p* = 0.80), except marginally shorter between the clustered and the dispersed treatments (21 [1.5, 45] %, *t* = 2.1, *p* = 0.085). There was no effect of spatial arrangement on eclosure ($\chi^{2}$(2) = 1.3, *p* = 0.52) and survival ($\chi^{2}$(2) = 0.48, *p* = 0.79), and the effects were not dependent on caterpillar size (eclosure_size × clusteredness_: $\chi^{2}$(2) = 0.37, *p* = 0.83; survival_size × clusteredness_: $\chi^{2}$(2) = 3.5, *p* = 0.17).

### 3.2 Proximal Causes of Differential Performance

Exploratory analyses of our structural hypotheses revealed that area of diet consumption, concentration of toxin ingested, step length (average caterpillar displacement), caterpillar pre‐weight and the interaction between proportion of time spent exploring and temporal variance experienced by the caterpillar underlie the differences in observed performance, explaining over three quarters of the variance (Figure 3). Importantly, the lack of support for direct paths between the variation treatment and RGR suggests that its treatment effect was completely mediated through these mechanisms. In contrast, these mechanisms explained roughly one‐third to one‐half of the treatment effect of clusteredness, with a moderate negative effect on RGR remaining unaccounted for.

As expected, greater movement, greater toxin ingestion and lower diet consumption reduced RGR (Figure 3). However, contrary to our hypotheses, spatial variance reduced movement and increased consumption, while clusteredness had no consistent effect on movement or toxin ingestion (Figure 3). Four other key results are noteworthy. First, increased consumption of diet for small caterpillars contributed most to the positive effect of variance treatment. Second, movement incurred a substantial cost on performance on par with the effect of increased toxin ingestion. Third, a moderate positive interaction between time spent exploring and temporal variance suggests that temporal variance suppressed herbivore performance when the caterpillar was not prone to exploration but enhanced it when the caterpillar was prone to exploration. Fourth, a caterpillar size‐dependent effect of clusteredness (Figure 4B,D) appears to be explained by changes in the inclination to explore and time spent on the more toxic diet. We proceeded to investigate the latter mechanism in more detail with different individual‐based models.

### 3.3 Comparison of Salient Behavioural Rules

**FIGURE 5:**
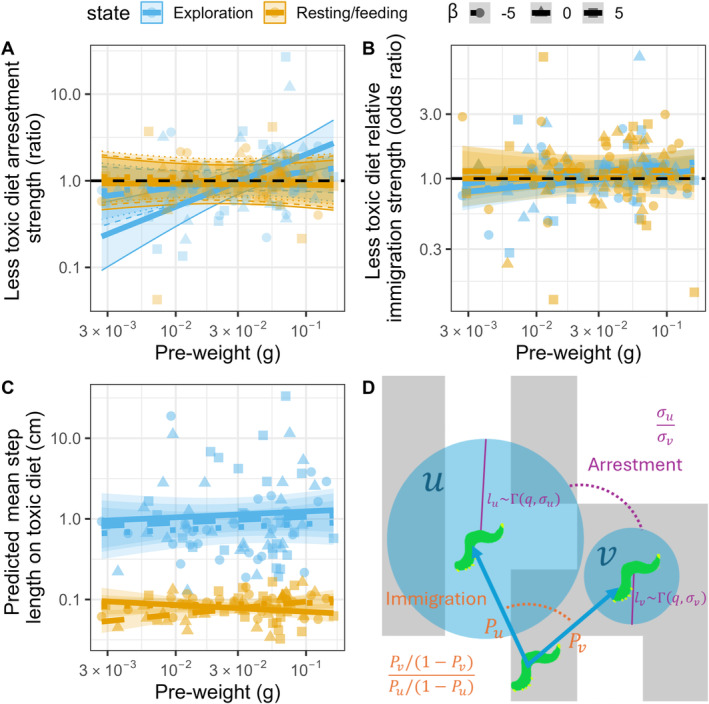
(A–C) Maximum likelihood estimates (points) of movement and behavioural parameters of each caterpillar taken from individually fitted integrated step selection functions. Separate parameters were estimated for each behavioural state (exploring or resting/feeding). Group means and 95% confidence intervals are displayed as lines and ribbons. (A, B) No preference is displayed as the black dashed line. (A) Only caterpillars in the clustered diet treatment group (β=5) in their exploration state displayed non‐zero and size‐dependent (significantly negative and positive) relative arrestment on the less toxic diet. (B) Only large caterpillars in the exploration state showed a significantly positive immigration towards a less toxic diet. (C) Caterpillars of different sizes moved the same average distances within each behavioural state. (D) A conceptual illustration of the different parameters shown in (A–C). Diet preference can be expressed through immigration (movements towards favoured diets) or arrestment (reductions in movement) (Appendices S9, S11). Assuming points *u* and *v* are equally available and accessible to a caterpillar at the bottom of the figure, relative immigration quantifies the odds ratio of the probability P of moving onto point *v* relative to point *u*. At points *u* and *v*, the caterpillar may move by a step length l, which is described by two gamma distributions, in the next time step. Arrestment quantifies how much less the caterpillar moves at *v* relative to *u* as the ratio of the scale parameter σ of the gamma distributions. All possible locations the caterpillars can move to in the next time step are shown as blue circles.

Further exploratory analyses revealed that caterpillars displayed systematic differences in behaviour depending on the body size, clusteredness treatment and behavioural state, but not the variation treatment (Table S1; Figure 5a–c). Notably, in the exploration state, the relative odds of moving onto a less toxic diet (immigration) increased marginally with caterpillar size (11 [−0.76, 25] %/SD, but see appendices 11, 14 for a caveat). In the clustered treatment, reduction in movement (arrestment) on the less toxic diet also increased with caterpillar size (85 [13, 203] % / SD). Small caterpillars significantly preferred to stay on more toxic diets ($\beta_{5}$: −80 [−93, −48] %), but not move onto more toxic diets (−18 [−38, 8.2] %). Large caterpillars significantly preferred to move onto (25 [0.20, 56] %) and stay on less toxic diets ($\beta_{5}$: 237 [66, 586] %). However, once caterpillars settled and entered the feeding/resting state, their movements over much shorter distance (~1/10th of the exploration state) were essentially random with respect to diet toxicity and did not increase in distance when the body size was larger.

Armed with these tentative behavioural observations, we used a simulation to verify which minimum set of behavioural assumptions can reproduce the size‐dependent effect of clusteredness on the proportion of time spent on the more toxic diet (Figure 3, Figure S6). Our *in silico* experiment revealed that discrimination between diet toxicities in the form of either increased movement into (immigration) or reduction in movement out of (arrestment) less toxic diets is necessary to generate differences between clusteredness treatments (Figure S5). That caterpillars show no diet preference at the feeding/resting state (Table S1) suggests movements in this state are mostly irrelevant to the effect of clusteredness. At the scale of movement in the exploration state, both immigration and arrestment can independently reproduce our observed qualitative effect of clusteredness. That is, clusteredness increased time spent on the more toxic diet for small caterpillars but decreased time spent on the more toxic diet for big caterpillars (Figure 5a,b). However, arrestment has a much larger effect size than that of immigration and can reproduce the magnitude on par with those observed in our study, especially when changes in arrestment were limited to the clustered treatment. When both immigration and arrestment occur simultaneously, the effect of immigration is reversed, attenuating the effect of arrestment. Taken together, these results support the hypothesis that how much caterpillars were arrested on the less toxic diets in the exploration state (Figure 5a) is primarily responsible for the interactive effect of clusteredness and body size.

## 4 Discussion

At all spatial scales, there is substantial variation in the amounts of phytochemicals that herbivores may encounter (Shelton 2005). The variation and spatial distribution of plant toxins is hypothesised to be a major determinant of where herbivores feed and how successful they are (Wetzel and Whitehead 2020; Zucker 1982), but little is known about how phytochemicals affect herbivores beyond the effect of the mean concentration. Likewise, prevailing theories of plant defence make no predictions about the fine‐scale pattern of toxin allocation beyond mean concentrations (McKey 1974; Rhoades 1979). We show that nontrivial emergent effects of xanthotoxin spatial variation and arrangement on herbivore movement, feeding and environmental experience can substantially affect the performance of herbivores. These observed effects are comparable in magnitude with that of mean xanthotoxin concentration (Pearse, Paul, and Ode 2018), in line with other modelling studies on heterogeneous distribution of stressors (Sears and Angilletta 2015). For illustration, for small caterpillars, reducing the variance from 2 to 0.5 mg^2^/g^2^ or changing the xanthotoxin arrangement from dispersed to clustered has a roughly equivalent effect on caterpillar growth as increasing the mean xanthotoxin concentration from 0.5 to 2 mg/g (Pearse, Paul, and Ode 2018). Therefore, fine‐scale variation in phytochemicals, a scale finer than most measurements of phytochemistry, can alter or even decouple the effects of mean phytochemical level measured from the whole plant or organ. Such decoupling is problematic because whole‐plant phytochemistry is a major tool in predicting host use patterns (Segar et al. 2017) and changes in plant‐herbivore interactions under climate change (Holopainen et al. 2018). Accordingly, the general lack of explanatory power of phytochemistry in predicting herbivory (Carmona, Lajeunesse, and Johnson 2011; Zvereva, Castagneyrol, and Kozlov 2024) may result from a mismatch in the scale of measurement and the micro‐scale at which herbivore experience plant traits.

Notably, our study demonstrates that these emergent effects of fine‐scale spatial heterogeneity could have arisen through interactions with simple foraging behaviours of herbivores. A randomly feeding herbivore does not benefit from greater spatial variance, nor is it affected by different spatial arrangements of toxins (Table 1). It is with behaviours specific to toxin concentrations that the average environment experienced by an herbivore deviates from the average environment. Depending on the specific behaviour and pattern of environmental heterogeneity, such deviation from the average condition can be to the benefit or detriment of the herbivore. Namely, with the appropriate behaviour, herbivores may compensate for high plant toxicity. Meanwhile, with certain spatial patterns of toxin allocation, plants may also make herbivores experience more toxins than the average concentration they produced per unit tissue (Figures S5, S6). Interestingly, our result that the effect of temporal variance depends on the exploration behaviour of the herbivore contrasts with previous work on the same system that found that temporal variation depressed herbivore performance via physiological mechanisms (Pearse, Paul, and Ode 2018). One plausible explanation may be that those caterpillars that explored more benefitted from the greater variety of food encountered to choose from. Our results thus highlight that temporal variation and spatial variation have distinct effects on herbivores through different mechanisms and that both effects are contingent upon the behaviour of the herbivore.

**TABLE 1:** Marginal effect of clusteredness ($p_{\beta_{5}}-p_{\beta_{-5}}$) on proportion of time spent p by caterpillars on the more toxic diet under different behavioural models with different degrees of realism (same data as in Figure S5).

Arrestment	Immigration	Size	Predicted effects	Overlap
No arrestment	No immigration	Small	−0.0020 [−0.18, 0.18]	0.23
		Large	−0.0020 [−0.18, 0.18]	0.13
Size‐dependent arrestment	No immigration	Small	0.14 [0.019, 0.26]	0.44
		Large	−0.098 [−0.22, 0.078]	0.28
No arrestment	Size‐dependent immigration	Small	0.019 [−0.17, 0.19]	0.26
		Large	−0.011 [−0.18, 0.15]	0.15
Size‐dependent arrestment	Size‐dependent immigration	Small	0.13 [0.034, 0.25]	0.38
		Large	−0.075 [−0.19, 0.085]	0.18
Size‐dependent arrestment in clustered treatment	No immigration	Small	0.23 [0.10, 0.37]	0.62
		Large	−0.38 [−0.50, −0.19]	0.58
Size‐dependent arrestment in clustered treatment	Size‐dependent immigration	Small	0.21 [0.11, 0.33]	0.53
		Large	−0.33 [−0.44, −0.17]	0.68

*Note:* We used $l∼\Gamma \left(q=0.8,\sigma =1\;\mathrm{cm}\right)$ for the step length distribution on the more toxic diet and $\theta ∼\mathrm{GvM}\left(\kappa_{1}=0.4,\kappa_{2}=0.3\right)$ for the turn angle distribution. The range of arrestment strength (reductions in movement) and immigration strength (movement towards favoured diets) correspond to the observed magnitude in Figure 5a,b. ‘No immigration’ or ‘no arrestment’ set the immigration and arrestment to 1, respectively. ‘Size dependent immigration’ and ‘size dependent arrestment’ set immigration and arrestment to 0.80 and 0.25 for small caterpillars, respectively, and 1.25 and 4 for large caterpillars, respectively. ‘Size dependent arrestment in clustered treatment’ sets arrestment to 0.25 and 4 for small and large caterpillars in the clustered treatment, respectively, and 1 for all caterpillars in other treatments. Predicted effects show mean and 95% quantiles of the simulations. Overlap shows the overlap coefficient (OVL) between the distribution of simulated predicted effect and the posterior distribution of observed effect (mean [95% credible intervals]; small = 0.28 [−0.025, 0.56]; large = −0.29 [−0.48, −0.099], Figure S6). Small and large caterpillars represent ±2 standard deviations of the range of sizes used in the experiment.

### 4.1 Push‐Pull Foraging in a Spatially Variable Environment

A key finding of our study is that herbivores used higher arrestment on less toxic diets to avoid feeding on more toxic diets (Figure 3; Figure S5, Appendix S14). Consequently, they circumvented most of the toxins available in the landscape, especially as the spatial variance in toxin concentration increased. This result is consistent with previous models that allowed herbivores to choose among heterogeneous resources (Shelton 2000; Thiel et al. 2021). However, contrary to those models and our prediction, the cost of diet preference did not increase with higher spatial variance. Rather, those that benefitted most from diet choice moved the least overall and did not suffer a considerable metabolic cost of movement (Figure 3). Such a reduction in movement was explained by the greater proportion of time spent on the less toxic diet where herbivores were arrested. Thus, whereas immigration incurs a cost of preference by having to move to preferred diets, arrestment on preferred diets does not incur such costs, at least in the form of movement that we could measure. Models that include herbivore choice in the form of immigration or repulsion (increases in movement), rather than arrestment, may therefore generate different qualitative predictions (Sears and Angilletta 2015; Shelton 2004).

Using simulations, we found other examples where different forms of diet preference produced different outcomes. Only arrestment, not diet immigration, could reproduce the observed effects of clusteredness (Table 1). For instance, for large caterpillars, arrestment combined with indiscriminate immigration often resulted in feeding spilling over to diet tiles near the initial diet tile they started on. Under the dispersed spatial regime, those nearby tiles were often more toxic than the initial tile they were arrested on, whereas under the clustered spatial regime, those nearby tiles were often of the same concentration. The spatial arrangement of phytochemicals varies considerably among natural systems due to vascular architecture, microenvironmental differences, developmental stochasticity and optimal defence (Figure 1; Jimenez‐Gomez et al. 2011; Orians and Jones 2001; Shroff et al. 2008). These different spatial regimes can encode different potential for risk and reward and likely contribute to the risk management strategies of herbivores, including dispersal, toxin tolerance and slow development, as have been discovered in seeds (Venable and Brown 1988).

Many plant toxins act on herbivore arrestment rather than immigration, especially when no long‐range cues can be used to detect their presence (Thorsteinson 1960). Younger larval insects generally have weak external olfactory capabilities and may rely more on gustatory cues to discriminate among diets (Wang et al. 2023). As such, they may use ‘test bites’ on random selections of potential feeding sites to decide if further feeding at the same site is warranted (Tsuneto et al. 2020). If it is generally true that the feeding choices of herbivores are expressed through arrestment rather than immigration, then some interesting predictions follow. Rather than making choices by comparing alternative food sources for which the quality is known, herbivores compare their current food source with their future expected food source. In a variable environment in which the future is uncertain, theory suggests that herbivores may exhibit successive contrast effects (McNamara, Fawcett, and Houston 2013), where past experiences alter the strength of preference towards the same quality diet (we demonstrate this phenomenon in Appendix S14). An herbivore that experiences a string of unfavourable diets under clustered spatial regimes may therefore settle, as an unfavourable environment is all that it knows. When the preferred food is rare, arrestment alone without immigration tends to be a poor strategy to spend more time on the preferred food, as the herbivore is unlikely to randomly move onto the preferred food. Therefore, the expression of diet preference exclusively through arrestment may be more common among diet generalists that can consume a variety of tissues or among herbivores in habitats where acceptable food is abundant.

## 5 Conclusion

Simple foraging behaviours of insect herbivores can have nontrivial emergent interactions with fine‐scale heterogeneity in phytochemistry, a scale relevant to the foraging behaviour of herbivores, but which is often overlooked in studies of phytochemistry. Importantly, both spatial variance and arrangement of phytochemistry affected the performance and space use of herbivores and did so at a magnitude that rivalled the effects of mean phytochemical concentration. Understanding the patterns and effects of intraspecific and intra‐individual phytochemical variation beyond the mean is still in its infancy. While many empirical and theoretical challenges remain, the stunning ubiquity and diversity of phytochemical patterns in nature (Figure 1) invite our investigation.

## Author Contributions

V.S.P., E.G., P.J.O., W.C.W. and I.S.P. conceived of the study. V.S.P., E.G., P.J.O. and I.S.P. collected the data. V.S.P. analysed the data and wrote the manuscript. All authors provided editorial support and approved the manuscript.

## Conflicts of Interest

The authors declare no conflicts of interest.

### Peer Review

The peer review history for this article is available at https://www.webofscience.com/api/gateway/wos/peer‐review/10.1111/ele.70044.

## Supporting information


Data S1.

File S1.

## Data Availability

The cleaned fitness, herbivory and movement data used in downstream analyses are deposited on Dryad (https://doi.org/10.5061/dryad.xksn02vqb). The code for data processing and analyses, as well as two custom R packages written for this project, are deposited on Zenodo (https://doi.org/10.5281/zenodo.12553862). It also contains all the raw data, intermediary data products (e.g. Mask‐R‐CNN predictions, movement tracks, image annotations and demo time lapse photos) and the code and data for the two supplemental experiments (Appendices 13 and 14). The version history of the code can be found on GitHub (https://github.com/vsbpan/spat_1f_noise and https://github.com/vsbpan/herbivar).

## References

[ele70044-bib-0001] Avgar, T. , J. R. Potts , M. A. Lewis , and M. S. Boyce . 2016. “Integrated Step Selection Analysis: Bridging the Gap Between Resource Selection and Animal Movement.” Methods in Ecology and Evolution 7: 619–630.

[ele70044-bib-0002] Barbosa, P. , J. Hines , I. Kaplan , H. Martinson , A. Szczepaniec , and Z. Szendrei . 2009. “Associational Resistance and Associational Susceptibility: Having Right or Wrong Neighbors.” Annual Review of Ecology, Evolution, and Systematics 40: 1–20.

[ele70044-bib-0003] Barthelme, S. , D. Tschumperle , J. Wijffels , et al. 2024. “Imager: Image Processing Library Based on ‘CImg’.”

[ele70044-bib-0004] Berenbaum, M. 1978. “Toxicity of a Furanocoumarin to Armyworms: A Case of Biosynthetic Escape From Insect Herbivores.” Science 201: 532–534. 17790440 10.1126/science.201.4355.532

[ele70044-bib-0005] Berenbaum, M. R. , and A. R. Zangerl . 1986. “Variation in Seed Furanocoumarin Content Within the Wild Parsnip ( *Pastinaca sativa* ).” Phytochemistry 25: 659–661.

[ele70044-bib-0006] Bernays, E. 2001. “Neural Limitations in Phytophagous Insects: Implications for Diet Breadth and Evolution of Host Affiliation.” Annual Review of Entomology 46: 703–727. 10.1146/annurev.ento.46.1.703 11112184

[ele70044-bib-0007] Bradski, G. 2000. “The OpenCV Library.” Dr Dobbs Journal 25: 120–125.

[ele70044-bib-0008] Brooks, M. E. , K. Kristensen , K. J. van Benthem , et al. 2017. “glmmTMB Balances Speed and Flexibility Among Packages for Zero‐Inflated Generalized Linear Mixed Modeling.” R Journal 9: 378–400.

[ele70044-bib-0009] Carmona, D. , M. J. Lajeunesse , and M. T. J. Johnson . 2011. “Plant Traits That Predict Resistance to Herbivores.” Functional Ecology 25: 358–367.

[ele70044-bib-0010] Cuddington, K. M. , and P. Yodzis . 1999. “Black Noise and Population Persistence.” Proceedings of the Royal Society of London ‐ Series B: Biological Sciences 266: 969–973.

[ele70044-bib-0011] Denny, M. 2015. Ecological Mechanics: Principles of Life's Physical Interactions. Princeton, New Jersey: Princeton University Press.

[ele70044-bib-0012] Fang, X.‐K. , D.‐F. Huang , Z.‐X. Wang , et al. 2007. “Identification of the Proteins Related to Cytochrome P450 Induced by Fenvalerate in a *Trichoplusia ni* Cell Line.” Cell Biology and Toxicology 23: 445–457. 17484068 10.1007/s10565-007-9006-1

[ele70044-bib-0013] Free Software Foundation, Inc . 2022. “GCC 12.2 manuals‐GNU Project.” Accessed June 24, 2024, https://gcc.gnu.org/onlinedocs/12.2.0/.

[ele70044-bib-0014] Gajewski, Z. , P. McElmurray , J. Wojdak , et al. 2024. “Nonrandom Foraging and Resource Distributions Affect the Relationships Between Host Density, Contact Rates and Parasite Transmission.” Ecology Letters 27: e14385. 38480959 10.1111/ele.14385

[ele70044-bib-0015] Gisiger, T. 2001. “Scale Invariance in Biology: Coincidence or Footprint of a Universal Mechanism?” Biological Reviews 76: 161–209. 11396846 10.1017/s1464793101005607

[ele70044-bib-0016] Gleadow, R. M. , and I. E. Woodrow . 2000. “Temporal and Spatial Variation in Cyanogenic Glycosides in *Eucalyptus cladocalyx* .” Tree Physiology 20: 591–598. 12651423 10.1093/treephys/20.9.591

[ele70044-bib-0017] Gonzalez, A. , and R. D. Holt . 2002. “The Inflationary Effects of Environmental Fluctuations in Source–Sink Systems.” Proceedings of the National Academy of Sciences 99: 14872–14877. 10.1073/pnas.232589299 PMC137511 12417745

[ele70044-bib-0018] Halley, J. M. 1996. “Ecology, Evolution and 1 f ‐Noise.” Trends in Ecology & Evolution 11: 33–37. 21237757 10.1016/0169-5347(96)81067-6

[ele70044-bib-0019] Hauri, K. C. , A. E. Glassmire , B. Randall , L. N. Zehr , and W. C. Wetzel . 2022. “Plant Chemical Diversity and Its Frequency Have Distinct but Complementary Effects on Insect Foraging.” Journal of Applied Ecology 59: 1362–1371.

[ele70044-bib-0020] Hauri, K. C. , A. E. Glassmire , and W. C. Wetzel . 2021. “Chemical Diversity Rather Than Cultivar Diversity Predicts Natural Enemy Control of Herbivore Pests.” Ecological Applications 31: e02289. 33423331 10.1002/eap.2289

[ele70044-bib-0021] Herrera, C. M. 2009. Multiplicity in Unity: Plant Subindividual Variation and Interactions With Animals. Interspecific Interactions: University of Chicago Press, Chicago, IL.

[ele70044-bib-0022] Holopainen, J. K. , V. Virjamo , R. P. Ghimire , J. D. Blande , R. Julkunen‐Tiitto , and M. Kivimäenpää . 2018. “Climate Change Effects on Secondary Compounds of Forest Trees in the Northern Hemisphere.” Frontiers in Plant Science 9: 1445. 30333846 10.3389/fpls.2018.01445 PMC6176061

[ele70044-bib-0023] Jimenez‐Gomez, J. M. , J. A. Corwin , B. Joseph , J. N. Maloof , and D. J. Kliebenstein . 2011. “Genomic Analysis of QTLs and Genes Altering Natural Variation in Stochastic Noise.” PLoS Genetics 7: e1002295. 21980300 10.1371/journal.pgen.1002295 PMC3183082

[ele70044-bib-0024] Karban, R. , A. A. Agrawal , and M. Mangel . 1997. “The Benefits of Induced Defenses Against Herbivores.” Ecology 78: 1351–1355.

[ele70044-bib-0025] Kendal, W. S. , and B. Jørgensen . 2011. “Tweedie Convergence: A Mathematical Basis for Taylor's Power Law, 1/f Noise, and Multifractality.” Physical Review E 84: 066120. 10.1103/PhysRevE.84.066120 22304168

[ele70044-bib-0026] Kennedy, J. S. 1978. “The Concepts of Olfactory ‘Arrestment’ and ‘Attraction.’.” Physiological Entomology 3: 91–98.

[ele70044-bib-0027] Lefcheck, J. S. 2016. “piecewiseSEM: Piecewise Structural Equation Modelling in r for Ecology, Evolution, and Systematics.” Methods in Ecology and Evolution 7: 573–579.

[ele70044-bib-0028] Legendre, P. 1993. “Spatial Autocorrelation: Trouble or New Paradigm?” Ecology 74: 1659–1673.

[ele70044-bib-0029] Lenth, R. V. , B. Bolker , P. Buerkner , et al. 2024. “Emmeans: Estimated Marginal Means, Aka Least‐Squares Means.”

[ele70044-bib-0030] Lewis, M. A. 1994. “Spatial Coupling of Plant and Herbivore Dynamics: The Contribution of Herbivore Dispersal to Transient and Persistent “Waves” of Damage.” Theoretical Population Biology 45: 277–312.

[ele70044-bib-0031] McKey, D. 1974. “Adaptive Patterns in Alkaloid Physiology.” American Naturalist 108: 305–320.

[ele70044-bib-0032] McNamara, J. M. , T. W. Fawcett , and A. I. Houston . 2013. “An Adaptive Response to Uncertainty Generates Positive and Negative Contrast Effects.” Science 340: 1084–1086. 23723234 10.1126/science.1230599

[ele70044-bib-0033] Nathan, R. , C. T. Monk , R. Arlinghaus , et al. 2022. “Big‐Data Approaches Lead to an Increased Understanding of the Ecology of Animal Movement.” Science 375: eabg1780. 35175823 10.1126/science.abg1780

[ele70044-bib-0034] Orians, C. M. , and C. G. Jones . 2001. “Plants as Resource Mosaics: A Functional Model for Predicting Patterns of Within‐Plant Resource Heterogeneity to Consumers Based on Vascular Architecture and Local Environmental Variability.” Oikos 94: 493–504.

[ele70044-bib-0035] Orians, C. M. , J. Pomerleau , and R. Ricco . 2000. “Vascular Architecture Generates Fine Scale Variation in Systemic Induction of Proteinase Inhibitors in Tomato.” Journal of Chemical Ecology 26: 471–485.

[ele70044-bib-0036] Pearse, I. S. , R. Paul , and P. J. Ode . 2018. “Variation in Plant Defense Suppresses Herbivore Performance.” Current Biology 28: 1981–1986.e2. 29887306 10.1016/j.cub.2018.04.070

[ele70044-bib-0037] Perret, D. L. , M. E. K. Evans , and D. F. Sax . 2024. “A species' Response to Spatial Climatic Variation Does Not Predict Its Response to Climate Change.” Proceedings of the National Academy of Sciences 121: e2304404120. 10.1073/pnas.2304404120 PMC10769845 38109562

[ele70044-bib-0038] Pincebourde, S. , and H. A. Woods . 2020. “There Is Plenty of Room at the Bottom: Microclimates Drive Insect Vulnerability to Climate Change.” Current Opinion in Insect Science 41: 63–70. 32777713 10.1016/j.cois.2020.07.001

[ele70044-bib-0039] Potter, K. A. , H. Arthur Woods , and S. Pincebourde . 2013. “Microclimatic Challenges in Global Change Biology.” Global Change Biology 19: 2932–2939. 23681970 10.1111/gcb.12257

[ele70044-bib-0040] R Core Team . 2023. R: A Language and Environment for Statistical Computing. Vienna, Austria: R Foundation for Statistical Computing.

[ele70044-bib-0041] Rhoades, D. F. 1979. “Evolution of Plant Chemical Defense Against Herbivores.” In Herbivores, Their Interaction With Secondary Plant Metabolites, edited by G. A. Rosenthal and D. H. Janzen , 3–54. New York: Academic Press.

[ele70044-bib-0042] Riolo, M. A. , P. Rohani , and M. D. Hunter . 2015. “Local Variation in Plant Quality Influences Large‐Scale Population Dynamics.” Oikos 124: 1160–1170.

[ele70044-bib-0043] Root, R. B. 1973. “Organization of a Plant‐Arthropod Association in Simple and Diverse Habitats: The Fauna of Collards ( *Brassica Oleracea* ).” Ecological Monographs 43: 95–124.

[ele70044-bib-0044] Ruokolainen, L. , A. Lindén , V. Kaitala , and M. S. Fowler . 2009. “Ecological and Evolutionary Dynamics Under Coloured Environmental Variation.” Trends in Ecology & Evolution 24: 555–563. 19699550 10.1016/j.tree.2009.04.009

[ele70044-bib-0045] Salazar, D. , and R. J. Marquis . 2022. “Testing the Role of Local Plant Chemical Diversity on Plant–Herbivore Interactions and Plant Species Coexistence.” Ecology 103: e3765. 35611398 10.1002/ecy.3765

[ele70044-bib-0046] Schmidt, K. A. , F. Massol , and J. Szymkowiak . 2022. “Resurrecting Shannon's Surprise: Landscape Heterogeneity Complements Information Use and Population Growth.” Oikos 2022: e09305.

[ele70044-bib-0047] Schultz, J. C. 1983a. “Habitat Selection and Foraging Tactics of Caterpillars in Heterogeneous Trees.” In Variable Plants and Herbivores in Natural and Managed Systems, edited by R. F. Denno and M. S. McClure , 61–90. New York: Academic Press.

[ele70044-bib-0048] Schultz, J. C. 1983b. “Impact of Variable Plant Defensive Chemistry on Susceptibility of Insects to Natural Enemies.” In Plant Resistance to Insects, ACS Symposium Series, 37–54. Washington, DC: American Chemical Society.

[ele70044-bib-0049] Sears, M. W. , and M. J. Angilletta . 2015. “Costs and Benefits of Thermoregulation Revisited: Both the Heterogeneity and Spatial Structure of Temperature Drive Energetic Costs.” American Naturalist 185: E94–E102. 10.1086/680008 25811092

[ele70044-bib-0050] Segar, S. T. , M. Volf , B. Isua , et al. 2017. “Variably Hungry Caterpillars: Predictive Models and Foliar Chemistry Suggest How to Eat a Rainforest.” Proceedings of the Royal Society B: Biological Sciences 284: 20171803. 10.1098/rspb.2017.1803 PMC5698651 29118136

[ele70044-bib-0051] Shelton, A. 2004. “Variation in Chemical Defences of Plants May Improve the Effectiveness of Defence.” Evolutionary Ecology Research 6: 709–726.

[ele70044-bib-0052] Shelton, A. L. 2000. “Variable Chemical Defences in Plants and Their Effects on Herbivore Behaviour.” Evolutionary Ecology Research 2: 231–249.

[ele70044-bib-0053] Shelton, A. L. 2005. “Within‐Plant Variation in Glucosinolate Concentrations of *Raphanus sativus* Across Multiple Scales.” Journal of Chemical Ecology 31: 1711–1732. 16222804 10.1007/s10886-005-5922-9

[ele70044-bib-0054] Shipley, B. 2009. “Confirmatory Path Analysis in a Generalized Multilevel Context.” Ecology 90: 363–368. 19323220 10.1890/08-1034.1

[ele70044-bib-0055] Shroff, R. , F. Vergara , A. Muck , A. Svatoš , and J. Gershenzon . 2008. “Nonuniform Distribution of Glucosinolates in *Arabidopsis thaliana* Leaves Has Important Consequences for Plant Defense.” Proceedings of the National Academy of Sciences 105: 6196–6201. 10.1073/pnas.0711730105 PMC2329684 18408160

[ele70044-bib-0056] Sutherland, D. W. S. , and G. L. Greene . 1984. “Cultivated and Wild Host Plants.” In Suppression and Management of Cabbage Looper Populations, 1–13. Technical Bulletin: U.S. Department of Agriculture.

[ele70044-bib-0057] Therneau, T. M. 2020. “Survival: Survival Analysis.” https://CRAN.R‐project.org/package=survival.

[ele70044-bib-0058] Thiel, T. , S. Gaschler , K. Mody , N. Blüthgen , and B. Drossel . 2020. “Impact of Plant Defense Level Variability on Specialist and Generalist Herbivores.” Theoretical Ecology 13: 409–424.

[ele70044-bib-0059] Thiel, T. , S. Gaschler , K. Mody , N. Blüthgen , and B. Drossel . 2021. “Impact of Herbivore Preference on the Benefit of Plant Trait Variability.” Theoretical Ecology 14: 173–187.

[ele70044-bib-0060] Thorsteinson, A. J. 1960. “Host Selection in Phytophagous Insects.” Annual Review of Entomology 5: 193–218.

[ele70044-bib-0061] Tsuneto, K. , H. Endo , F. Shii , K. Sasaki , S. Nagata , and R. Sato . 2020. “Diet Choice: The Two‐Factor Host Acceptance System of Silkworm Larvae.” PLoS Biology 18: e3000828. 32936797 10.1371/journal.pbio.3000828 PMC7494105

[ele70044-bib-0062] Underwood, N. , B. D. Inouye , and P. A. Hambäck . 2014. “A Conceptual Framework for Associational Effects: When Do Neighbors Matter and How Would we Know?” Quarterly Review of Biology 89: 1–19. 24672901 10.1086/674991

[ele70044-bib-0063] Van Rossum, G. , and F. L. Drake . 2009. Python 3 Reference Manual. Scotts Valley, CA: CreateSpace.

[ele70044-bib-0064] Vasseur, D. A. , and P. Yodzis . 2004. “The Color of Environmental Noise.” Ecology 85: 1146–1152.

[ele70044-bib-0065] Venable, D. L. , and J. S. Brown . 1988. “The Selective Interactions of Dispersal, Dormancy, and Seed Size as Adaptations for Reducing Risk in Variable Environments.” American Naturalist 131: 360–384.

[ele70044-bib-0066] Wang, Q. , H. M. Smid , M. Dicke , and A. Haverkamp . 2023. “The Olfactory System of *Pieris brassicae* Caterpillars: From Receptors to Glomeruli.” Journal of Insect Science 31: 469–488. 10.1111/1744-7917.13304 38105530

[ele70044-bib-0067] Warton, D. I. , and F. K. C. Hui . 2011. “The Arcsine Is Asinine: The Analysis of Proportions in Ecology.” Ecology 92: 3–10. 21560670 10.1890/10-0340.1

[ele70044-bib-0068] Wetzel, W. C. , B. D. Inouye , P. G. Hahn , S. R. Whitehead , and N. Underwood . 2023. “Variability in Plant–Herbivore Interactions.” Annual Review of Ecology, Evolution, and Systematics 54: 451–474.

[ele70044-bib-0069] Wetzel, W. C. , H. M. Kharouba , M. Robinson , M. Holyoak , and R. Karban . 2016. “Variability in Plant Nutrients Reduces Insect Herbivore Performance.” Nature 539: 425–427. 27749815 10.1038/nature20140

[ele70044-bib-0070] Wetzel, W. C. , and J. S. Thaler . 2016. “Does Plant Trait Diversity Reduce the Ability of Herbivores to Defend Against Predators? The Plant Variability‐Gut Acclimation Hypothesis.” Current Opinion in Insect Science 14: 25–31. 27436643 10.1016/j.cois.2016.01.001

[ele70044-bib-0071] Wetzel, W. C. , and S. R. Whitehead . 2020. “The Many Dimensions of Phytochemical Diversity: Linking Theory to Practice.” Ecology Letters 23: 16–32. 31724320 10.1111/ele.13422

[ele70044-bib-0072] Whitham, T. G. 1981. “Individual Trees as Heterogeneous Environments: Adaptation to Herbivory or Epigenetic Noise?” In Insect Life History Patterns, edited by R. F. Denno and H. Dingle , 9–27. New York, NY: Springer.

[ele70044-bib-0073] Woods, H. A. , M. E. Dillon , and S. Pincebourde . 2015. “The Roles of Microclimatic Diversity and of Behavior in Mediating the Responses of Ectotherms to Climate Change. *J. Therm. Biol*., What Sets the Limit?” How Thermal Limits, Performance and Preference in Ectotherms Are Influenced by Water or Energy Balance 54: 86–97. 10.1016/j.jtherbio.2014.10.002 26615730

[ele70044-bib-0074] Yang, Q. , M. S. Fowler , A. L. Jackson , and I. Donohue . 2019. “The Predictability of Ecological Stability in a Noisy World.” Nature Ecology & Evolution 3: 251–259. 30697002 10.1038/s41559-018-0794-x

[ele70044-bib-0075] Zangerl, A. R. , J. G. Hamilton , T. J. Miller , et al. 2002. “Impact of Folivory on Photosynthesis Is Greater Than the Sum of Its Holes.” Proceedings of the National Academy of Sciences 99: 1088–1091. 10.1073/pnas.022647099 PMC117434 11792866

[ele70044-bib-0076] Zucker, W. V. 1982. “How Aphids Choose Leaves: The Roles of Phenolics in Host Selection by a Galling Aphid.” Ecology 63: 972–981.

[ele70044-bib-0077] Zvereva, E. L. , B. Castagneyrol , and M. V. Kozlov . 2024. “Does Spatial Variation in Insect Herbivory Match Variations in Plant Quality? A Meta‐Analysis.” Ecology Letters 27: e14440. 38778587 10.1111/ele.14440

