# Supplementary material for: Large Differences in Herbivore Performance Emerge From Simple Herbivore Behaviours and Fine‐Scale Spatial Heterogeneity in Phytochemistry
Source: Ecol Lett. 2024 Dec 31;28(1):e70044. doi: 10.1111/ele.70044 (PMC11687352; doi:10.1111/ele.70044)
Supplement: Supplementary file 1 — Data S1. [file ELE-28-0-s002.pdf]

Ecology Letters

Large Differences in Herbivore Performance Emerge from Simple Herbivore Behaviors and Fine-Scale Spatial Heterogeneity in Phytochemistry

Vincent S. Pan[1-3,9], Enakshi Ghosh[4], Paul J. Ode[4-5], William C. Wetzel[1-3,6], Kadeem J. Gilbert[1-3,7], Ian S. Pearse[8]

1: Department of Integrative Biology, Michigan State University, East Lansing, MI, USA

2: W. K. Kellogg Biological Station, Michigan State University, Hickory Corners, MI, USA

3: Ecology, Evolution, and Behavior Program, Michigan State University, Easting Lansing, MI, USA

4: Department of Agricultural Biology, Colorado State University, Fort Collins, CO, USA

5: Graduate Degree Program in Ecology, Colorado State University, Fort Collins, CO, USA

6: Land Resources and Environmental Sciences, Montana State University, Bozeman, Montana, USA

7: Department of Plant Biology, Michigan State University, East Lansing, MI, USA

8: U.S. Geological Survey, Fort Collins Science Center, Fort Collins, CO, USA

9: Corresponding author: vsbpan@gmail.com

Disclaimer statement

Any use of trade, firm, or product names is for descriptive purposes only and does not imply endorsement by the U.S. Government.

APPENDIX 1: Experimental arena

We constructed thirty experimental arenas out of 20 × 20 × 8 cm clear plastic containers in which we hot glued a 12 × 12 × 1 cm wooden square at the center (Figure 2a). The plastic dome helps to maintain humidity, thereby preventing the artificial diet from drying out. A single Raspberry Pi camera was mounted 15 cm above the plastic lid with a piece of wire. At the beginning of each round of experiment, we lined the bottom of the wooden square with a ~ 18 × 18 cm layer of plastic cling film. This further reduced moisture loss and prevented the diet raster from falling apart when it was moved to a second container without a camera. The transparent bottom of the arenas allowed us to individually place artificial diet tiles according to a printed template of the assigned diet pattern which we placed beneath the plastic container. After assembling the 144 diet tiles into a full 12 × 12 raster, we placed two 11.5 × 1 × 0.5 cm wooden strips inside the wooden square, but underneath the cling film to squeeze the diet tiles in place. This addition effectively eliminated any gaps between individual diet tiles. We used an airbrush to spray a thin layer of red luster dust (Red Food Grade Metallic Luster Dust, Inoceris) on the artificial diet to facilitate the computer in distinguishing between eaten and uneaten parts of the diet. Finally, after a caterpillar was put inside the wooden square, we covered the square with a 12.5 × 12.5 cm plate of glass to ensure the caterpillar could not escape. We sprayed antifog (Fog Defender System Anti-Fog Spray, Zeiss) on the underside of the glass plate to prevent condensation buildup over the course of the experiment.

To prepare diet tiles used in the study, we prepared 600 ml of 0, 0.5, 1.5, 2 mg / g diet and 300 ml of 1 mg / g diet. For every liter of diet, we added 162 g of general noctuid diet powder (Southland Products Inc., Lake Village, Arkansas) to 930 ml of boiling deionized water. We also added 5 ml of linseed oil to facilitate proper adult wing development and xanthotoxin

(8-methoxypsoralen, CAS 298-81-7, Sigma-Aldrich) according to treatment. To create diet tiles, we poured the hot diet into 1 × 1 × 1 cm ice cube trays then cut individual cubes into 1 × 1 × 0.5 cm tiles with a wire cheese cutter. We stored the prepared diet at 4°C.

A detailed analysis of the performance and demographic response of *Trichoplusia ni* to the same concentrations of xanthotoxin (with and without variation) we used are reported in Pearse *et al.* (2018) and Paul *et al.* (2021). In both studies, increasing mean concentration of xanthotoxin was found to reduce caterpillar weight gain and increase development time, and Pearse *et al.* (2018) showed a decrease in female fecundity and intrinsic population growth rate. With a different range of concentrations, Akhtar & Isman (2003) found that increasing xanthotoxin concentration caused reduced feeding. Likewise, Dussourd (2003) found that increasing xanthotoxin concentration caused leaf abandonment in *T. ni*, and at no concentration did it induce trenching behavior.

APPENDIX 2: *Trichoplusia ni* colony

The *Trichoplusia ni* colony was obtained from both Jena Johnson (University of Georgia, Department of Entomology) and Benzon Research (Carlisle, Pennsylvania, USA). All caterpillars used in the study started as eggs laid by adults from the colony. We reared these insects in groups of 2-3 caterpillars per individual cup half-filled with 2.5 ml of xanthotoxin free artificial diet. We kept these caterpillars in incubators set at a 16/8 day/night cycle at 25°C, occasionally moving some caterpillars to 15°C to slow their development to synchronize enough individuals for the next round of experiments. We washed the eggs and pupae with 0.5% bleach to suppress mold growth. Adults were fed a 10% honey water solution.

APPENDIX 3: Spectral synthesis

To generate randomized diet landscapes that exhibit different degrees of clusteredness belonging to the family of $\frac{1}{f^{\beta}}$ power law noises, we used a two-dimensional extension of the spectral synthesis method from Cuddington & Yodzis (1999). First, we generated amplitudes A_{f_r} of a given spectral frequency f in the u and v directions, following

$$A_{f_r} = \frac{\epsilon_{f_r}}{f_r^{\frac{\beta}{2}}}, \qquad (S1)$$

$$\epsilon \sim N(0,1), \qquad (S2)$$

$$f_r = \sqrt{f_u^2 + f_v^2}, \qquad (S3)$$

where β is the chosen spectral exponent that determines the color of the noise and ϵ is a gaussian random variable that is modified by the power law to create random amplitudes. We then generated the phase ϕ of each frequency from a uniform distribution that gives all directions of oscillation equal probability.

$$\phi \sim U(0,2\pi) \qquad (S4)$$

With the randomly generated amplitudes and phases, we performed inverse Discrete Fast Fourier Transformation in the u and v directions to arrive at randomly generated values in the spatial domain.

$$h(x,y) = \mathcal{F}_u^{-1}[\mathcal{F}_v^{-1}[A_{f_r}\exp(i\phi_{f_r})]] \qquad (S5)$$

Because our treatment only allows for binary values, for each generated raster $h(x,y)$, we created a binary raster by thresholding by the median value. We added a very small random value $a \sim N(0, 0.00001)$ to each cell of the raster to avoid ties in thresholding.

APPENDIX 4: Caterpillar monitoring

To measure where caterpillars move and feed throughout their larval stage, we attached a Raspberry Pi Zero W computer equipped with an 8-MegaPixel Pi Camera (Adafruit Industries, New York, New York) without an infrared filter on top of each arena. The cameras took a 1,400 × 1,400-pixel RGB image every six minutes throughout the experimental duration, ending when either five days had elapsed, the caterpillar began to spin silk, or when the caterpillar had died. In total, we captured 150,230 images with an average of 1,008 images per trial. Because the caterpillars did not move quickly in our trial experiments, we deemed this sampling frequency sufficient to capture most of the movement exhibited by a caterpillar. With each time lapse image, we performed a quad-to-quad transformation to crop the diet landscape into a perspective corrected 1,000 × 1,000-pixel image for downstream analyses (Figure 2a). This transformation was done by rearranging pixels from the original image into a destination image using the following relation,

$$\begin{bmatrix} q_x \\ q_y \end{bmatrix} = \begin{bmatrix} (a_1 p_x + a_2 p_y + a_3)/(1 + a_7 p_x + a_8 p_y) \\ (a_4 p_x + a_5 p_y + a_6)/(1 + a_7 p_x + a_8 p_y) \end{bmatrix}, \qquad \text{(S6)}$$

where $\begin{bmatrix} q_x \\ q_y \end{bmatrix}$ is the coordinate of the destination, $\begin{bmatrix} p_x \\ p_y \end{bmatrix}$ is the coordinate of the origin, and $a_1, \ldots, a_8$ are coefficients found by solving the following linear system,

$$\begin{bmatrix} p_{1,x} & p_{1,y} & 1 & 0 & 0 & 0 & -p_{1,x}q_{1,x} & -p_{1,y}q_{1,x} \\ 0 & 0 & 0 & p_{1,x} & p_{1,y} & 1 & -p_{1,x}q_{1,y} & -p_{1,y}q_{1,y} \\ p_{2,x} & p_{2,y} & 1 & 0 & 0 & 0 & -p_{2,x}q_{2,x} & -p_{2,y}q_{2,x} \\ 0 & 0 & 0 & p_{2,x} & p_{2,y} & 1 & -p_{2,x}q_{2,y} & -p_{2,y}q_{2,y} \\ p_{3,x} & p_{3,y} & 1 & 0 & 0 & 0 & -p_{3,x}q_{3,x} & -p_{3,y}q_{3,x} \\ 0 & 0 & 0 & p_{3,x} & p_{3,y} & 1 & -p_{3,x}q_{3,y} & -p_{3,y}q_{3,y} \\ p_{4,x} & p_{4,y} & 1 & 0 & 0 & 0 & -p_{4,x}q_{4,x} & -p_{4,y}q_{4,x} \\ 0 & 0 & 0 & p_{4,x} & p_{4,y} & 1 & -p_{4,x}q_{4,y} & -p_{4,y}q_{4,y} \end{bmatrix} \begin{bmatrix} a_1 \\ a_2 \\ a_3 \\ a_4 \\ a_5 \\ a_6 \\ a_7 \\ a_8 \end{bmatrix} = \begin{bmatrix} q_{1,x} \\ q_{1,y} \\ q_{2,x} \\ q_{2,y} \\ q_{3,x} \\ q_{3,y} \\ q_{4,x} \\ q_{4,y} \end{bmatrix}. \qquad \text{(S7)}$$

Here, $\begin{bmatrix} p_{1,x} \\ p_{1,y} \end{bmatrix}, \ldots, \begin{bmatrix} p_{4,x} \\ p_{4,y} \end{bmatrix}$ are the vertices of the origin quadrilateral and $\begin{bmatrix} q_{1,x} \\ q_{1,y} \end{bmatrix}, \ldots, \begin{bmatrix} q_{4,x} \\ q_{4,y} \end{bmatrix}$ are the vertices of the destination quadrilateral. The transformation was done using the backward algorithm with linear interpolation from the package *imager* ver. 0.42.13 (Barthelme *et al.* 2024). We manually picked the coordinates of the four vertices of the diet landscape as the origin using a custom R shiny app (*shiny* ver. 1.7.1 Chang *et al.* 2024). We recorded the movement of 120 / 128 individual caterpillars. Seven trials were conducted without an active camera, and the images of one trial were corrupted by a USB drive that failed.

To extract caterpillar movement and feeding information from the cropped images, we used a custom trained Mask-Region-based Convolutional Neural Network (Mask-R-CNN) to identify the pixels belonging to the caterpillar body and keypoints corresponding to specific body parts of the caterpillar (head, middle, and posterior of the caterpillar, Figure 2a). The model was implemented using the *PyTorch* (ver. 2.1.1 with *CUDA* 12.1 support, Paszke *et al.* 2019) interface *detectron2* ver. 0.6 (Wu *et al.* 2019). We used a ResNet-50 and Feature Pyramid Network backbone with initial weights pre-trained on the Common Objects in Context 2017 dataset for 37 epochs. We trained the model for five epochs on a randomly sampled set of ~27 images per replication (including ten trial replications) with camera footage. We manually annotated each image for three keypoints and a polygon for the caterpillar using COCO Annotator (Brooks 2019). To speed up the annotation process, a first pass annotation was predicted from a prototype Mask-R-CNN model trained on a smaller dataset, but each annotation was manually verified or corrected. A total of 4,070 images were annotated, of which 85% (3,459 images) was used for training, 5% was used for validation (i.e., tuning model hyperparameters; 204 images), and 10% was used for testing (i.e., evaluating model performance; 407 images).

To improve learning, we implemented random image augmentations, including spatial transformations (rotations, and horizontal and vertical flips) and color scale transformations (brightness, saturation, contrast, and lighting adjustments). To identify the head of the caterpillar in each image, we kept only the highest scoring Region of Interest (RoI) with a confidence score greater than 0.7 corresponding to a caterpillar. Each RoI is then used to create a heatmap of the likelihood for each keypoint. We sequentially updated the heatmap after identifying the most confident keypoint, thus preventing the model from confusing the posterior of the caterpillar with the head.

For keypoints detection, our model achieved a precision of 91% and recall of 79% on the testing dataset, and a precision of 93% and recall of 84% on the training dataset (appendix 5). For caterpillar mask detection, our model achieved a precision of 91% and recall of 79% on a testing dataset, and a precision of 91% and recall of 82% on the training dataset (appendix 5). We further validated the neutral network predictions using three automated error flagging methods that detect anomalies in repeated long-distance jumps to the same location, implausible caterpillar mask size, and repeated switching between posterior and head keypoints (appendix 6). Finally, upon reviewing the cleaned movement tracks, we removed nine trials that still had poor detections. After removing flagged errors, we retained 83% detection for the median trial (*mean* = 74%, *95% quantiles* = [14%, 95%]). Roughly 5% of the total timesteps had failed detection because the caterpillar was outside of frame. In total, we acquired 105,587 valid time steps for downstream analyses (mean = 760 / trial, *95% quantiles* = [46 / trial, 1,127 / trial]). An example movement sequence prediction outcome is shown in Video S1.

APPENDIX 5: Mask R-CNN inference evaluation

To assess how well our custom Mask-R-CNN model predicted keypoints and binary masks, we consider two primary challenge metrics,

$$precision = \frac{TP}{TP + FP} , \tag{S8}$$

$$recall = \frac{TP}{TP + FN} , \tag{S9}$$

where TP is the number of true positive predictions, FP is the number of false positive predictions, and FN is the number of false negative predictions. Precision measures the proportion of detections generated by the model that are correct, whereas recall measures the proportion of true instances the model identifies.

To compute the number of positive and negative predictions requires a way of measuring the similarity between the predicted and ground truth instances. We considered the model predicted keypoints identical with the ground truth keypoints if it satisfies the inequality, a standard object keypoint similarity (OKS) metric,

$$OKS = \frac{\sum_i^N \exp\left(-\frac{d_i^2}{2s^2k^2}\right)}{N} \geq 0.5, \tag{S10}$$

where d_i is the Euclidean distance between two keypoints of type i (head, middle, or posterior), s^2 is the area of the ground truth caterpillar binary mask, k = 100, and N is the number of labeled keypoints. We determined the value of k by testing whether the metric was able to distinguish between correct predictions and errors in a test set of manually scored images. We considered the model predicted mask identical to the ground truth mask if it satisfies the inequality, a standard mask similarity metric,

$$IoU = \frac{A_i}{A_u} \geq 0.5, \tag{S11}$$

where A_i and A_u is the area of intersection and area of union between the predicted and ground truth masks.

APPENDIX 6: Mask-R-CNN inference post-processing

We implemented three automated methods to flag prediction errors from the neural network and remove those potential errors aggressively. In the first pass, we identified instances where there were repeated switches between the position of the keypoints assigned to the head and the posterior of the caterpillar, but the caterpillar was apparently remaining still. This error arises when the head of the caterpillar is at an obscured angle or when the model is not very confident of whether one end of the caterpillar is the posterior or anterior. As a result, the model flickers between correct and incorrect predictions between successive frames. To identify this error, we used a two-stage approach. First, we identified successive frames for which the caterpillar head moved by more than r_{thresh}, but the centroid of the caterpillar binary mask did not, where r_{thresh} is half of the square root of the median caterpillar binary mask size. Second, we took these suspicious frames and performed a similar, but more accurate assessment of whether the binary mask moved by testing whether the IoU between successive frames fall below 0.5. Frames that were also identified in this stage were removed from the cleaned data (*mean* = 12%, *95% quantiles* = [1.5%, 32%]).

In the second pass, we sought to identify cases where the caterpillar binary mask had an implausible size, which could indicate a misidentified caterpillar. To do so, for each frame, we computed the Z-score of the mask area within a three-hour window and identified whether the mask of the caterpillar was potentially partially obscured outside of the frame, defined as being within 20 pixels to the edge of the frame. Instances where the caterpillar was in frame and for

which the absolute Z-score was greater than two were removed from the cleaned data (*mean* = 2.4%, *95% quantiles* = [0.93%, 3.9%]).

In the third pass, we sought to identify cases where caterpillars made repeated long-distance movements to a single location. This error arises when the model mistakes debris or shadow as a caterpillar and flickers between correct and incorrect predictions throughout the timelapse. To detect this type of error, we took instances where the movement of the binary mask centroid was greater than 200 pixels and counted whether those movements were clustered at specific locations in 60 × 60-pixel bins. Since long distance movements are rare, long-distance movements that start exactly in the same location multiple times are very unlikely. Instances where the caterpillar was identified within the spatial bin with the highest count were removed from the cleaned data. We applied this third error flagging method only to selected replications for which we found anomalous movement patterns in a plot of the raw movement tracks (*mean* = 1.0%, *95% quantiles* = [0.0%, 6.9%]).

APPENDIX 7: Herbivory detection

To quantify how much and what kind of diet the caterpillars consumed over the course of the experiment, we analyzed the final frame of the time lapse (Figure S2a) to derive an herbivory binary mask (Figure S2f). We used this binary mask to estimate the area of diet eaten and computed a weighted average of the concentration of xanthotoxin eaten,

$$\bar{Q} = \frac{\sum_i w_i Q_i}{\sum_i w_i} \qquad (S12)$$

where w_i is the number of pixels of the eaten diet belonging to the diet type i with toxin concentration Q_i.

To derive the binary mask, we first took the RGB image and computed the hue index of the image using,

$$Hue = \text{atan}\left(\frac{2(B-G-R)}{30.5}(G-R)\right) \qquad (S13)$$

as defined by Olivoto (2022), where the B, G, and R are the color channel intensities (Figure S2b). We subtracted the hue of the third image (12 minutes into the experiment) from the hue of the final image to ensure that damages to the diet present at the beginning of the experiment are not counted. We also set the region 20 pixels around the binary mask prediction of the caterpillar as the 10th quantile of the image hue difference to ensure that the caterpillar is not mistaken as eaten diet (Figure S2c). We then enhanced the contrast of the image by solving the screened Poisson equation (package *imagerExtra*, Ochi *et al.* 2019) using $\lambda = 0.001$ to correct for inhomogeneous background (Figure S2d), before performing binary thresholding using Otsu's method (Figure S2e). Finally, we performed morphological erosion on the binary mask to remove the speckles (false detections) in the final binary mask (Figure S2f).

APPENDIX 8: Behavioral segmentation

An examination of the raw caterpillar movement tracks (Figure 2) reveals at least two distinct modes of movement. One mode of movement corresponds to an exploration state, where the caterpillar makes successive long-distance movements as it searches for an appropriate feeding site. A second mode of movement corresponds to a feeding/resting state, where the caterpillar makes very short distance movements as it feeds or rests at a chosen feeding site. Segmenting these two modes of movement is necessary for appropriate estimation of movement parameters of and realistic simulation of different movement models. To this end, for each movement track of an individual caterpillar (the sequence of step lengths and turn angles), we

fitted a two-state Hidden Markov Model (HMM) where we estimated a state transition matrix and state specific step length and turn angle distribution parameters (*moveHMM* ver. 1.9 Michelot *et al.* 2016). We used a lognormal distribution and wrapped Cauchy distribution for the step length and turn angle distributions respectively. We labeled the longest step taken by each caterpillar as the exploration state. The behavioral state of the rest of the timepoints were inferred from the fitted HMM using the Viterbi algorithm.

APPENDIX 9: Estimation of Movement and Behavioral Parameters

To determine systematic differences in behavioral patterns between treatments and to parameterize our individual-based movement models, we performed integrated Step Selection Analysis (iSSA) on the individual movement track of each caterpillar (Avgar *et al.* 2016). Briefly, an integrated step selection model estimates simultaneously, a movement free habitat selection kernel and a selection free movement kernel, comprising of a turn angle distribution and step length distribution (Figure 2b). This estimation is done by performing conditional logistic regression (stratified by step identity) on the true step and a set of 'available steps' simulated from the movement kernel and corresponding habitat covariates at each time step (package *amt* ver. 0.2.1.0, Signer *et al.* 2019, package *survival* ver. 3.3-1, Therneau 2020). By 'true step', we mean a step that we observed the caterpillar actually making. By 'available steps' (*sensu* Avgar *et al.* 2016), we mean steps within reach of the caterpillar that it could have taken at a given time point. Movement into closer patches has a higher probability. Movement to outside of the experimental arena has a probability of zero.

To fit an integrated step selection function (iSSF), we first used maximum likelihood fits of gamma distribution and generalized von Mises distribution as the tentative step length and

turn angle kernels to generate tentative available steps (Figure 2c). We included log step length and step length to estimate the shape and scale parameters of the gamma distribution (Appendix S2 eqn. 2.3.1 of Avgar *et al.* 2016). We included $\cos(\theta + \pi)$ and $\cos(2\theta)$, where θ is the turn angle, to estimate the parameters of the generalized von Mises distribution (eqn. S16, appendix 9). Coefficients associated with these movement characteristics were used to update the tentative step length and turn angle distributions, such that the estimations of these distributions take habitat preference into account and become selection free (Avgar *et al.* 2016). We allowed different step length and turn angle kernel parameters to be estimated for each behavioral state (exploration or resting/feeding). In each model we fitted to each individual movement track, we allowed the step length and turn angle kernel parameters to further vary between when the caterpillar is on a more toxic or less toxic diet (i.e., we estimated a total of four turn angle and four step length distributions). We also included a binary predictor denoting whether a step ends up on a less toxic diet a covariate and allowed it to interact with behavioral state. Doing so allows us to estimate the movement free selection strength of the less toxic diet (commonly known as 'relative selection strength' or RSS, but which we refer to as 'immigration'). At each step, we simulated 100 available steps and removed those that were outside of the experimental arena. Trials with fewer than thirty valid observations per behavioral state were removed. Overall, we were able to estimate the movement parameters for n = 75 - 81 caterpillars, depending on the parameter.

We compared three behavioral rules between treatments: (i) differential arrestment on the less toxic diet relative to the more toxic diet, (ii) differential immigration onto a less toxic diet relative to a more toxic diet, and (iii) different scales of movement. By 'arrestment', we mean orthokinesis; that is, slowing or stopping of locomotion on a diet (*sensu* Kennedy 1978 and

reference therein). By ‘immigration’, we mean effective movement (taxis) towards a favored diet. It is also known as ‘attraction’ (Kennedy 1978), commonly measured through Y-tube experiments, and falls under a narrower definition of ‘relative selection strength’ (Avgar *et al.* 2016). We used the term ‘immigration’ to emphasize its phenomenological definition and that the process of diet selection occurs among locations where the insect can move to, through directed movement or indirect movements (e.g., via a series of steppingstones). Importantly, both arrestment and immigration are ways in which preference for a type of diet can be exhibited, at least effectively. This is because the amount of time an insect spends on a type of diet is governed by the balance between immigration into and emigration out of that type of diet. Arrestment is involved when an insect chooses between its current diet and some expected diet in future time. Immigration is involved when an insect chooses between available diets in space (Figure 2b, 5d). Both can occur simultaneously.

For (i), we computed the ratio between the scale parameter of the step length distributions on the more and less toxic diet. A higher value means that the caterpillar takes shorter step lengths on the less toxic diet. For (ii), we simply used the movement free relative selection strength for the less toxic diet. A higher value means that a caterpillar is more likely to move onto a less toxic diet all else being equal (but refer to appendix 11). For (iii), we computed the predicted mean step length as a product between the shape and scale parameter of the gamma distribution. We modeled the maximum likelihood estimate of each behavioral-state-specific-movement rule parameter of each caterpillar in six linear mixed models. Each movement parameter was log-transformed as they are strictly positive. In each model, we included clusteredness treatment, variance treatment, and caterpillar size as fixed effects, and

experimental session as a random intercept. We allowed caterpillar size to interact with treatments.

APPENDIX 10: Generalized von Mises Distribution in iSSA

Here, we suggest a new turn angle distribution for integrated step selection analysis (ISSA) that better accommodates our data. For the distribution of turn angles θ in our simulation of available steps, we consider a special case of the generalized von Mises distribution (GvM *sensu* Gatto & Jammalamadaka 2007) which has the density function,

$$P(\theta;\ \kappa_1, \kappa_2) = \frac{\exp(\kappa_1 \cos(\theta\ + \pi) + \kappa_2 \cos(2\theta))}{\int_0^{2\pi} \exp(\kappa_1 \cos(\theta\ + \pi) + \kappa_2 \cos(2\theta))\, \mathrm{d}\theta}. \qquad (S14)$$

The generalized von Mises distribution is an extension of the von Mises distribution, commonly used to model turn angles, that can account for bimodality. A second peak centered at 180° that represents backtracking can often be found in animal turn angle distributions (Figure S3). Although some alternative distributions to model such behavior have been proposed, such as the wrapped Cauchy distribution (Yackulic *et al.* 2011) and mixed von Mises distribution (Potts *et al.* 2014), neither lends itself easily to the modeling framework of iSSA. This is because iSSA involves updating a tentative turn angle distribution using regression coefficients from a conditional logistic regression. Doing so requires that the log likelihood function of distribution parameters be written as a linear function of some function of turn angles (Avgar *et al.* 2016). Indeed, it can be shown that the probability of observing a turn angle θ drawn from a generalized von Mises distribution can be expressed as an exponential function of some linear combination of $\cos(\theta + \pi)$ and $\cos(2\theta)$,

$$\ln P(\theta;\ \kappa_1, \kappa_2) \propto \kappa_1 \cos(\theta\ + \pi) + \kappa_2 \cos(2\theta)\,. \qquad (S15)$$

Thus, given the likelihood function eqn. S14, the maximum likelihood estimates of the regression coefficients α_{κ_1} and α_{κ_2} can be seen as an estimator of the bias of κ_1 and κ_2 in the tentative distributions respectively.

$$\prod_{t=3}^{T} \frac{\exp\left(\alpha_{\kappa_1}\cos(\theta_t + \pi) + \alpha_{\kappa_2}\cos(2\theta_t)\right)}{\sum_{\mathrm{i}=0}^{S}\exp\left(\alpha_{\kappa_1}\cos\left(\theta'_{i,t} + \pi\right) + \alpha_{\kappa_2}\cos\left(2\theta'_{i,t}\right)\right)} \quad \text{(S16)}$$

Here, T is a set of spatial positions with turn angle θ_t. θ'_i is an element of a set S of randomly generated available turn angles. Therefore, κ_1 and κ_2 can be updated using,

$$\widehat{\kappa_1} = \kappa_1 + \alpha_{\kappa_1} \quad \text{(S17.1)}$$
$$\widehat{\kappa_2} = \kappa_2 + \alpha_{\kappa_2} \quad \text{(S17.2)}$$

We estimated the maximum likelihood model parameter values using the probability density function defined in eqn. S14 and the Nelder-Mead method. Random draws from this distribution were generated using the standard ratio-of-uniforms algorithm from Gatto (2008).

APPENDIX 11: Validation of iSSF parameter identifiability

In this study, we outlined two distinct behavioral processes of preference (i.e., arrestment and immigration; Figure 5d). But while both arrestment and immigration are well defined and mutually exclusive under ideal scenarios, it remains to be seen whether these processes can be reliably estimated from data with violations to model assumptions. In particular, when the observation frequency of the movement of a caterpillar is much lower than the frequency at which the behavioral processes occur, the effect of arrestment and immigration may look identical and prevent accurate estimation.

To explore when parameter identifiability is an issue, we performed a simulation experiment in which we simulated movement tracks with only either the arrestment or

immigration process, set at a strength of 3. The distribution of toxin is generated from white noise using the same grain and size as those used in our real-life experiment. We drew step lengths from $l \sim \Gamma(q = 1, \sigma = 0.108 \text{ cm})$, where q is the shape parameter and σ is the scale parameter. We drew turn angles from $\theta \sim U(-\pi, \pi)$. To test if a lower sampling frequency prevents accurate estimation, for a subset of the simulations, we kept only every third observation (66% drop out). We fitted an iSSF to the first 2,000 observations of each movement track, estimating the arrestment and immigration parameters simultaneously. At each time step, we simulated 100 available steps. We repeated the simulation 200 times for each observation frequency and process scenarios.

Our simulation revealed that integrated step selection analysis can accurately estimate true values of arrestment and immigration when observations are collected at the same frequency at which the simulated process occurs (Figure S4). This result affirms that the two processes are mutually exclusive and well defined. However, sampling at 1/3rd the frequency (66% drop out), arrestment can masquerade as immigration, with deflated estimates of arrestment and inflated estimates of immigration, when there should be none (Figure S4). On the other hand, immigration does not masquerade as arrestment at low sampling frequencies, though the estimates of immigration are inflated (Figure S4). Taken together, these results suggest that iSSA cannot distinguish a pure arrestment process from a mixed arrestment and immigration process. While a significant effect of arrestment is an honest indication of the existence of the assessment process, a significant effect of immigration may arise from unobserved arrestments. Therefore, we conclude that arrestment is likely underestimated in our study, and we have insufficient evidence that large caterpillars make comparisons of toxicity among available diets and make movements toward their preferred diet.

APPENDIX 12: Behavioral rules *in silico* experiment

We simulated different individual-based models that include various combinations of behavioral rules under different spatial clusteredness regimes. We considered changing three behavioral rules within the range of observed values, including, (i) differential arrestment on the less toxic diet relative to the more toxic diet, (ii) differential preference towards moving onto a less toxic diet relative to a more toxic diet, and (iii) different scales of movement. To do so, we varied the less toxic diet immigration strength from 0.75 to 1.33. We fixed the turn angle distribution at $\theta \sim GvM(\kappa_1 = 0.4, \kappa_2 = 0.3)$ and the step length distribution at $l \sim \Gamma(q = 0.8, \sigma)$. We used different values of σ_{less} and σ_{more} depending on whether the virtual caterpillar is on a less or more toxic diet. We systematically varied σ_{more} from 0.1 cm to 1 cm, which roughly correspond to observed scale parameters in the feeding/resting and exploring states respectively. We also varied σ_{less} systematically relative to σ_{more} by an inverse factor of less toxic diet arrestment strength. Toggling the scale parameter of the gamma distribution is preferable to toggling the mean parameter because the distribution remains identical under rescaling. That is, given that l is a random variable generated from a gamma distribution with scale σ, for any scaling factor $c > 0$, the following holds,

$$cl \sim \Gamma(q, \sigma c). \qquad (S18)$$

Finally, for each movement rule combination (27 total), we simulated a virtual caterpillar foraging on a randomly generated virtual diet landscape with different degrees of clusteredness for 1,000 timesteps. We repeated this simulation 400 times (*n* = 32,400 total simulations), each time recording the proportion of time spent on the more toxic diet. We chose this pattern to analyze because it appeared to have some explanatory power for caterpillar RGR in our

exploratory analysis with a structural equation model. Results of the simulation are shown in Figure S5 and Table 1.

To estimate observed effect sizes to compare with our simulation, we fitted proportion time spent on the more toxic diet in a Bayesian Hierarchical Generalized Linear Mixed Model with a logit-linked beta conditional distribution (package *brms* ver. 2.19.0, Bürkner 2017). We included clusteredness and variance treatments, and caterpillar log pre-weight as fixed effects. Clusteredness was allowed to interact with caterpillar log pre-weight. Experimental session was included as a random intercept. We placed moderately regularizing priors on all estimated parameters. Results are shown in Figure S6. Effect sizes were calculated by computing $p_{\beta_5} - p_{\beta_{-5}}$ for each posterior draw for large and small caterpillars, where p is the proportion time spent on the more toxic diet. We used the overlap coefficient (package *bayestestR* ver. 0.13.1, Makowski *et al.* 2019) to quantify the similarity between the distribution of simulated predicted effect and the posterior distribution of the observed effect.

APPENDIX 13: Constant toxin concentration experiment

To test how no xanthotoxin variation compares with the low variation and high variation treatments in our main experiment, we conducted an additional experiment alongside our main experiment in which we placed caterpillars on a constant 1 mg / g xanthotoxin landscape using the same methods as before. We repeated this experiment twice per experimental session, totaling n = 10 (but only eight survived to pupation). We fitted RGR and time to pupation in two separate generalized linear mixed models with an identity linked normal and log linked gamma distribution respectively. We included session identity as a random effect and variation treatment, log caterpillar pre-weight, and their interactions as fixed effects. As before, we also

added a quadratic term for log caterpillar pre-weight due to the significant non-linearity detected. We did not include clusteredness because it is undefined for the constant treatment (there is no variation to be spatially arranged).

Overall, our analysis revealed that the constant treatment had similar effects on caterpillar performance as the low variation treatment (Figure S1a-b). As before, we found evidence that variation treatment and caterpillar pre-weight interactively affected caterpillar RGR ($\chi^2(2) = 10$, $P = 0.0067$), but not time to pupation ($\chi^2(2) = 0.71$, $P = 0.70$). Small caterpillars that fed on a constant 1 mg/g xanthotoxin diet exhibited a lower RGR than caterpillars that fed on a highly variable diet (0.012 $hour^{-1}$; 2.2 SD), although this difference was only marginally significant ([-4.2, -0.18] SD, $t = -2.1$, $P = 0.066$). Caterpillars in the constant treatment group had a non-significant lower RGR compared to caterpillars in the low treatment group (-0.75 [-2.8, 1.3] SD, $t = -0.72$, $P = 0.69$). For large caterpillars, there was no significant difference between the constant treatment group and any of the variable treatment groups (constant vs high: 0.88 [-0.88, 2.6] SD, $t = 0.98$, $P = 0.52$; constant vs low: 0.64 [-0.26, 1.5] SD, $t = 0.71$, $P = 0.69$). This attenuation of the effect of variance treatment could be because larger caterpillars tend to be less susceptible to toxins and already have a low baseline RGR, so there is less room for a reduction due to treatment. For time to pupation, there was a general trend towards longer pupation time the less variable the landscape was ($\chi^2(2) = 18.6$, $P < 0.0001$). Caterpillars in the constant treatment had a 27% longer time to pupation than caterpillars in the high variation treatment ([13, 45] %, $t = 3.8$, $P = 0.0003$), and a marginally 15% longer time to pupation than caterpillars in the low variation treatment ([1.2, 30] %, $t = 2.2$, $P = 0.059$).

Finally, using this dataset, we tested whether our results presented in the main text may be explained better by the presence/absence of toxin-free food in the high variance treatment as

opposed to the variance of the toxin concentration. If our interpretation is correct that variance in the toxin concentration is the primary driver, a model which includes the variance of the concentration of xanthotoxin should fit better than a model which includes the binary presence/absence of toxin-free food. To evaluate this prediction, we refitted our models for RGR and time to pupation in which we replaced the variation treatment predictor (previously coded as a factor) with either a variable denoting the binary presence/absence of toxin-free food or with a continuous variable for the variance of toxin concentration. We found that the model which includes variance fitted slightly better for RGR (ΔAICc = 0.50), and significantly better for time to pupation (ΔAICc = 2.4). Given that we can already find a significant difference with a small dataset, we conclude that toxin variance is a better explanation of our results than the binary presence/absence of toxin-free diet.

APPENDIX 14: Xanthotoxin conditioning experiment

We conducted an additional experiment to explicitly verify our conclusions that were borne out of our statistical analysis of movement tracks and individual based model simulations. Specifically, we aimed to test whether 1) *T. ni* can sense local, but not long range, xanthotoxin concentration and that 2) choice is made between prior experience and current conditions. A key prediction of the latter hypothesis is the presence of successive contrast effects (McNamara *et al.* 2013), where caterpillars that were reared on toxic diets are predicted to exhibit stronger preference towards toxic diets compared to caterpillars that were reared on toxin free diets. ‘Toxin conditioned' caterpillars prefer toxic diets more because their perception of future expected toxin availability is much higher than toxin naïve caterpillars.

To test these hypotheses, we reared 99 caterpillars on either a toxic (2 mg/g) or not toxic (0 mg/g) diet for one week and recorded in a 48-hour choice assay whether the caterpillars moved to find a new diet and if so, whether the diet was of a high or low xanthotoxin concentration (Figure S7). In each trial, we placed four toxic diets (2 mg/g) and one not toxic diet (0 mg/g) at five centimeter-intervals in a ring at a random order. A caterpillar was placed in the center of each ring so that it is equidistant from each diet. Half of the caterpillars started on a center block that was toxic (2 mg/g). We fitted the first-choice outcomes (low concentration, high concentration, or no choice) in a Bayesian generalized linear model with a logit-linked categorical conditional distribution. We included the center block treatment, the conditioning treatment, the weight of the caterpillar before the choice assay, and their interactions as predictors.

If the caterpillar moves randomly with respect to toxin concentration over long distances, we expect to see the first choice being the less toxic diet at a rate equal to the proportion of that diet in the environment (i.e., 25%). This prediction is confirmed for caterpillars reared on either toxic or toxin free diets and holds across all range of caterpillar sizes tested (Figure S8). This result is consistent with the fact that xanthotoxin, like all furanocoumarins, is non-volatile. As such, it is highly unlikely that foraging caterpillars can detect the presence or absence or even the concentrations of this toxin from a distance. We therefore conclude that there is no evidence that *T. ni* can sense the concentration of xanthotoxin itself over 5 cm in artificial diet.

Next, we may ask whether caterpillars use their past experience to evaluate the quality of the diet they are on. A prerequisite of this hypothesis, if true, is that the caterpillars can sense the concentration of toxin in the diet they are on. Therefore, we merely have to test the second hypothesis to confirm the first hypothesis. If our second hypothesis that caterpillars use their past

experience to evaluate the quality of their current diet is true, we may predict that toxin naïve caterpillars are more likely to move away from their center block diet if the diet that the caterpillars start on in the experiment is toxic. Our results are shown in Figure S9, confirming that caterpillars do indeed move away from the toxic center block based on prior toxin experience. This result is consistent with previous experiments from Akhtar & Isman (2004) who also showed that previous exposure to xanthotoxin increases feeding response of *T. ni* to xanthotoxin containing food. This result was not due to lower locomotion capacity of caterpillars reared on a toxic diet. Indeed, we may evaluate whether the locomotion capacity of the caterpillars is equal between conditioning treatments by comparing whether caterpillars reared on toxic or toxin-free diets made first choices at an equal rate (as opposed to not making a first choice and staying at the center of the ring). We find that caterpillars were equally capable of making a first choice when there was no center block (Figure S9). Taken together, we conclude that in so far as under our simplistic experimental condition, *T. ni* can sense the level of xanthotoxin in their artificial diet locally, but not over a distance, and move non-randomly with respect to fine-scale heterogeneity in toxin concentration based on their prior experience.

References

Akhtar, Y. & Isman, M.B. (2003). Binary mixtures of feeding deterrents mitigate the decrease in feedingdeterrent response to antifeedants following prolonged exposure in thecabbage looper, *Trichoplusia ni* (Lepidoptera: Noctuidae). *Chemoecology*, 13, 177–182.

Akhtar, Y. & Isman, M.B. (2004). Generalization of a Habituated Feeding Deterrent Response to Unrelated Antifeedants Following Prolonged Exposure in a Generalist Herbivore, Trichoplusia ni. *J Chem Ecol*, 30, 1349–1362.

Avgar, T., Potts, J.R., Lewis, M.A. & Boyce, M.S. (2016). Integrated step selection analysis: bridging the gap between resource selection and animal movement. *Methods Ecol. Evol.*, 7, 619–630.

Barthelme, S., Tschumperle, D., Wijffels, J., Assemlal, H.E., Ochi, S., Robotham, A., *et al.* (2024). imager: Image Processing Library Based on "CImg." https://cran.r-project.org/web/packages/imager/index.html

Brooks, J. (2019). COCO Annotator. version 0.11.1. https://github.com/jsbroks/coco-annotator/

Bürkner, P.-C. (2017). brms: An R Package for Bayesian Multilevel Models Using Stan. *J. Stat. Softw.*, 80, 1–28.

Chang, W., Cheng, J., Allaire, J.J., Sievert, C., Schloerke, B., Xie, Y., *et al.* (2024). shiny: Web Application Framework for R. https://cran.r-project.org/web/packages/shiny/index.html

Cuddington, K.M. & Yodzis, P. (1999). Black noise and population persistence. *Proc. R. Soc. Lond. B Biol. Sci.*, 266, 969–973.

Dussourd, D.E. (2003). Chemical Stimulants of Leaf-Trenching by Cabbage Loopers: Natural Products, Neurotransmitters, Insecticides, and Drugs. *J Chem Ecol*, 29, 2023–2047.

Gatto, R. & Jammalamadaka, S.R. (2007). The generalized von Mises distribution. *Stat. Methodol.*, 4, 341–353.

Gatto, R. (2008). Some computational aspects of the generalized von Mises distribution. *Stat. Comput.*, 18, 321–331.

Kennedy, J.S. (1978). The concepts of olfactory 'arrestment' and 'attraction.' *Physiol. Entomol.*, 3, 91–98.

Makowski, D., Ben-Shachar, M.S. & Lüdecke, D. (2019). bayestestR: Describing Effects and their Uncertainty, Existence and Significance within the Bayesian Framework. *J. Open Source Softw.*, 4, 1541.

McNamara, J.M., Fawcett, T.W. & Houston, A.I. (2013). An Adaptive Response to Uncertainty Generates Positive and Negative Contrast Effects. *Science*, 340, 1084–1086.

Michelot, T., Langrock, R. & Patterson, T.A. (2016). moveHMM: an R package for the statistical modelling of animal movement data using hidden Markov models. *Methods Ecol. Evol.*, 7, 1308–1315.

Ochi, S., Yu, G., Sapiro, G., Sbert, C., Line, I.P.O. & Getreuer, P. (2019). imagerExtra: Extra Image Processing Library Based on "imager." https://cran.r-project.org/web/packages/imagerExtra/index.html

Olivoto, T. (2022). Lights, camera, pliman! An R package for plant image analysis. *Methods Ecol. Evol.*, 13, 789–798.

Paszke, A., Gross, S., Massa, F., Lerer, A., Bradbury, J., Chanan, G., *et al.* (2019). PyTorch: An Imperative Style, High-Performance Deep Learning Library. *arXiv*, arXiv:1912.01703 http://arxiv.org/abs/1912.01703

Paul, R.L., Pearse, I.S. & Ode, P.J. (2021). Fine-scale plant defence variability increases top-down control of an herbivore. *Func. Ecol.*, 35, 1437–1447.

Pearse, I.S., Paul, R. & Ode, P.J. (2018). Variation in Plant Defense Suppresses Herbivore Performance. *Curr. Bio.*, 28, 1981-1986.e2.

Potts, J.R., Bastille-Rousseau, G., Murray, D.L., Schaefer, J.A. & Lewis, M.A. (2014). Predicting local and non-local effects of resources on animal space use using a mechanistic step selection model. *Methods Ecol. Evol.*, 5, 253–262.

Signer, J., Fieberg, J. & Avgar, T. (2019). Animal movement tools (amt): R package for managing tracking data and conducting habitat selection analyses. *Ecol. Evol.*, 9, 880–890.

Therneau, T.M. (2020). survival: Survival Analysis.

Wu, Y., Kirillov, A., Massa, F., Lo, W.-Y. & Girshick, R. (2019). Detectron2. https://github.com/facebookresearch/detectron2

Yackulic, C.B., Blake, S., Deem, S., Kock, M. & Uriarte, M. (2011). One size does not fit all: flexible models are required to understand animal movement across scales. *J. Anim. Ecol.*, 80, 1088–1096.

Table S1. Comparison of behavioral rule parameters (less toxic diet immigration strength, less toxic diet arrestment strength, mean step length on less toxic diet) between treatments and caterpillar body size. Main effects display test of refitted model coefficients when the nonsignificant interaction with body size is dropped. Group means display unique group means in units of ratio (immigration, arrestment) or centimeters (step length). 95% confidence intervals are shown in brackets. Significant interactions and main effects are bolded. Group means that are significantly different from the null are bolded. Small and large caterpillars are denoted with 'S' and 'L' respectively.

**Behavioral parameter**	**Behavioral state**	**Body size interaction**	**Main effects**	**Group means**
Immigration (odds ratio)	Exploring	Clusteredness: $\chi^2(2) = 0.22$, $P = 0.89$ Variance: $\chi^2(1) = 0.44$, $P = 0.51$	Clusteredness: $\chi^2(2) = 1.6$, $P = 0.45$ Variance: $\chi^2(1) = 0.15$, $P = 0.70$ Body size: $\chi^2(1) = 3.3$, $P = 0.067$	S: 0.82 [0.62, 1.1] **L: 1.3 [1.0, 1.6]**
Immigration (odds ratio)	Feeding/resting	Clusteredness: $\chi^2(2) = 3.9$, $P = 0.14$ Variance: $\chi^2(1) = 1.0$, $P = 0.31$	Clusteredness: $\chi^2(2) = 0.56$, $P = 0.75$ Variance: $\chi^2(1) = 0.031$, $P = 0.86$ Body size: $\chi^2(1) = 0.0024$ $P = 0.96$	1.1 [0.95, 1.3]
Arrestment (ratio)	Exploring	Clusteredness: $\boldsymbol{\chi^2(2) = 6.5, P = 0.038}$ Variance: $\chi^2(1) = 0.23$, $P = 0.63$	Clusteredness: $\chi^2(2) = 0.63$, $P = 0.73$ Variance: $\chi^2(1) = 0.022$, $P = 0.88$ **Body size:** $\boldsymbol{\chi^2(1) = 6.9\ P = 0.0088}$	Sβ_{-5}: 0.74 [0.33, 1.7] Sβ_0: 0.63 [0.27, 1.5] **S$\boldsymbol{\beta_5}$: 0.20 [0.073, 0.52]** Lβ_{-5}: 1.1 [0.55, 2.1] Lβ_0: 1.5 [0.71, 3.1] **L$\boldsymbol{\beta_5}$: 3.4 [1.7, 6.9]**
Arrestment (ratio)	Feeding/resting	Clusteredness: $\chi^2(2) = 0.40$, $P = 0.82$ Variance: $\chi^2(1) = 0.42$, $P = 0.52$	Clusteredness: $\chi^2(2) = 1.3$, $P = 0.52$ Variance: $\chi^2(1) = 3.7$, $P = 0.053$ Body size: $\chi^2(1) = 0.011$ $P = 0.92$	Low var.:0.88 [0.67, 1.2] High var.: 1.1 [0.85, 1.4]
Step length (cm)	Exploring	Clusteredness: $\chi^2(2) = 0.36$, $P = 0.83$ Variance: $\chi^2(1) = 0.57$, $P = 0.45$	Clusteredness: $\chi^2(2) = 1.4$, $P = 0.48$ Variance: $\chi^2(1) = 2.9$, $P = 0.090$ Body size: $\chi^2(1) = 0.35$ $P = 0.55$	**0.96 [0.75, 1.2]**
Step length (cm)	Feeding/resting	**Clusteredness:** $\boldsymbol{\chi^2(2) = 6.5, P = 0.039}$ Variance: $\chi^2(1) = 0.0088$, $P = 0.93$	Clusteredness: $\chi^2(2) = 3.0$, $P = 0.22$ Variance: $\chi^2(1) = 0.88$, $P = 0.35$ Body size: $\chi^2(1) = 0.19$ $P = 0.67$	**S$\boldsymbol{\beta_{-5}}$: 0.082 [0.058, 0.11]** **S$\boldsymbol{\beta_0}$: 0.051 [0.036, 0.073]** **S$\boldsymbol{\beta_5}$: 0.097 [0.064, 0.15]** **L$\boldsymbol{\beta_{-5}}$: 0.092 [0.069, 0.12]** **L$\boldsymbol{\beta_0}$: 0.11 [0.080, 0.14]** **L$\boldsymbol{\beta_5}$: 0.066 [0.049, 0.089]**

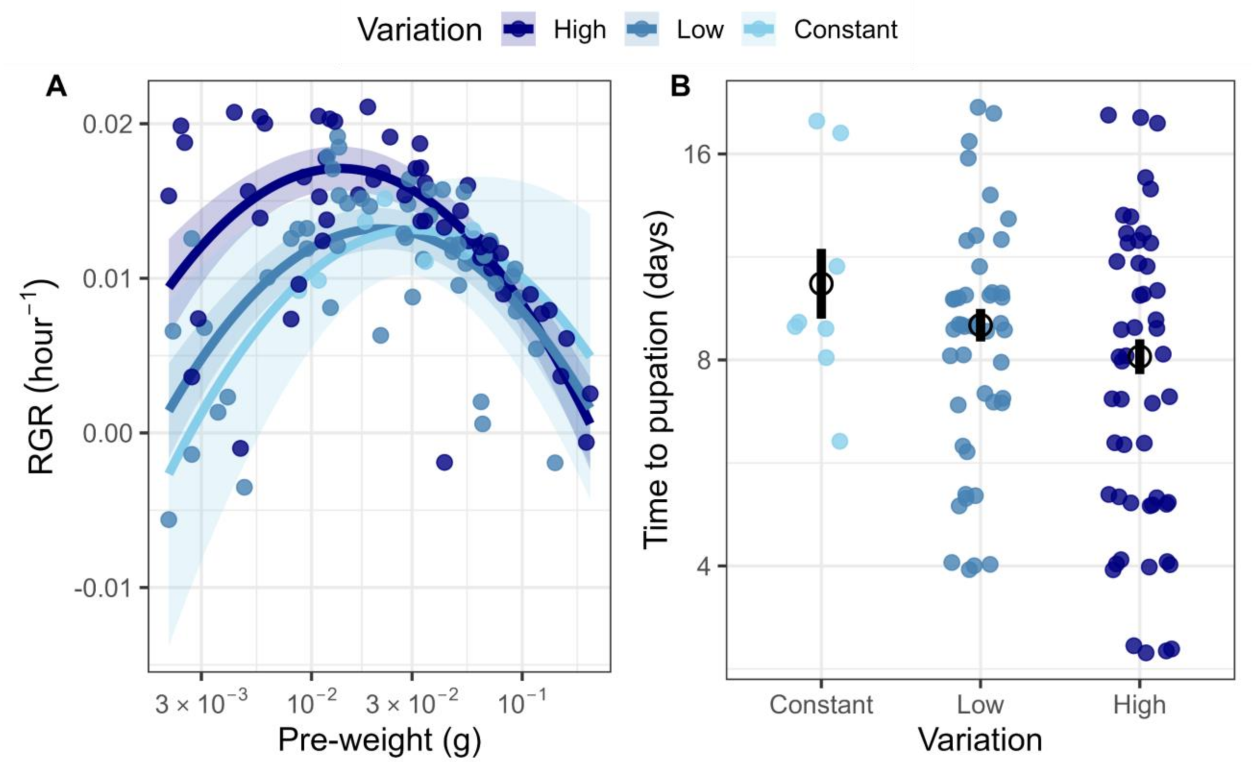

Figure S1. Caterpillar performance across variation treatment levels. Each point represents a caterpillar. Ribbons and error bars show 95% confidence intervals. Solid lines and open circles show mean estimates.

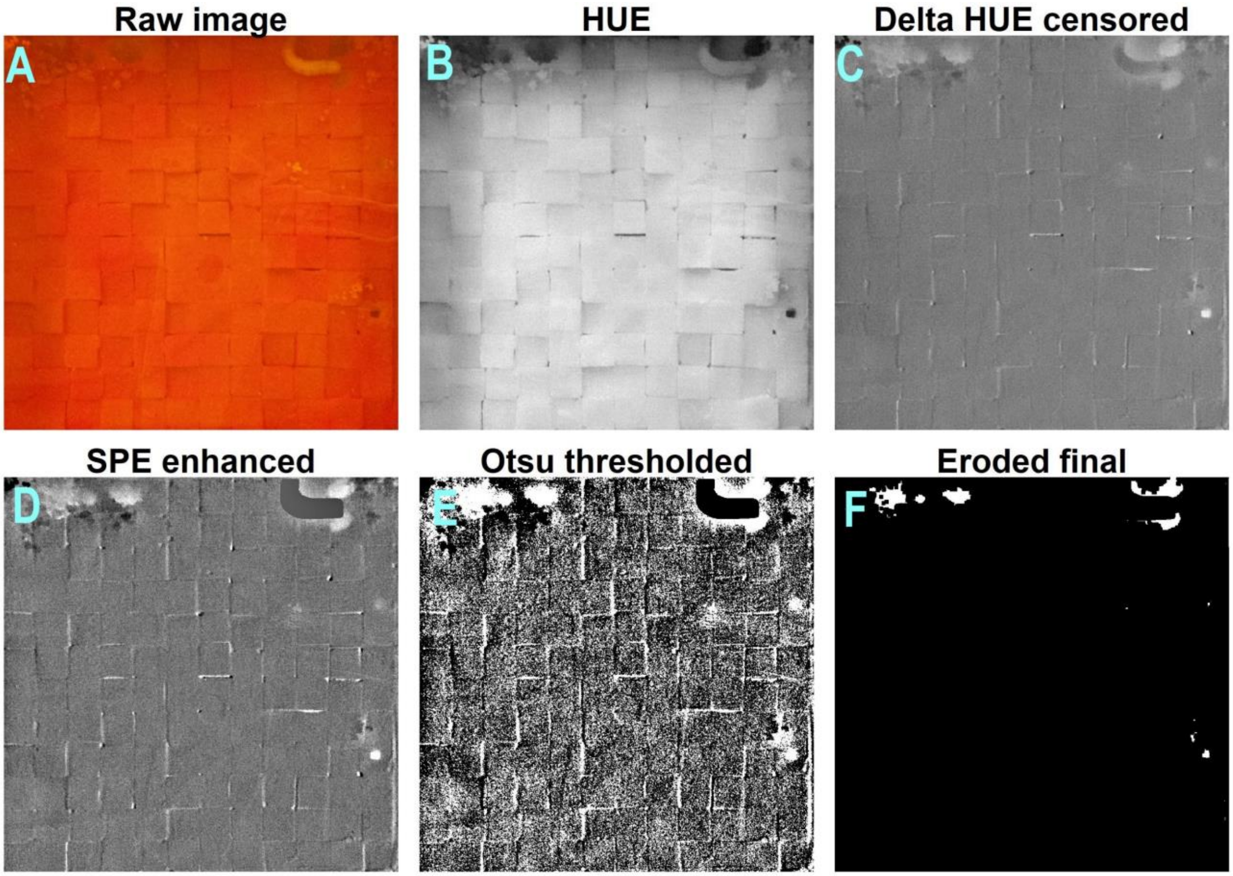

Figure S2. Steps in estimating diet consumption mask from an image captured by the Pi camera. (A) the original RGB image that has been cropped and perspective corrected via quad-to-quad transformation. A layer of red luster dust on the surface of the diet gives the uneaten parts a red coloration. (B) The color hue of A. (C) the difference in hue between B and the third frame. The widened caterpillar mask predicted by the Mask R-CNN is censored. (D) C after contrast enhancement via solving the screened Poisson equation. (E) Thresholded binary image of D via Otsu's method. (F) Binary mask erosion that cleans up the speckles in E, arriving at the final consumption mask.

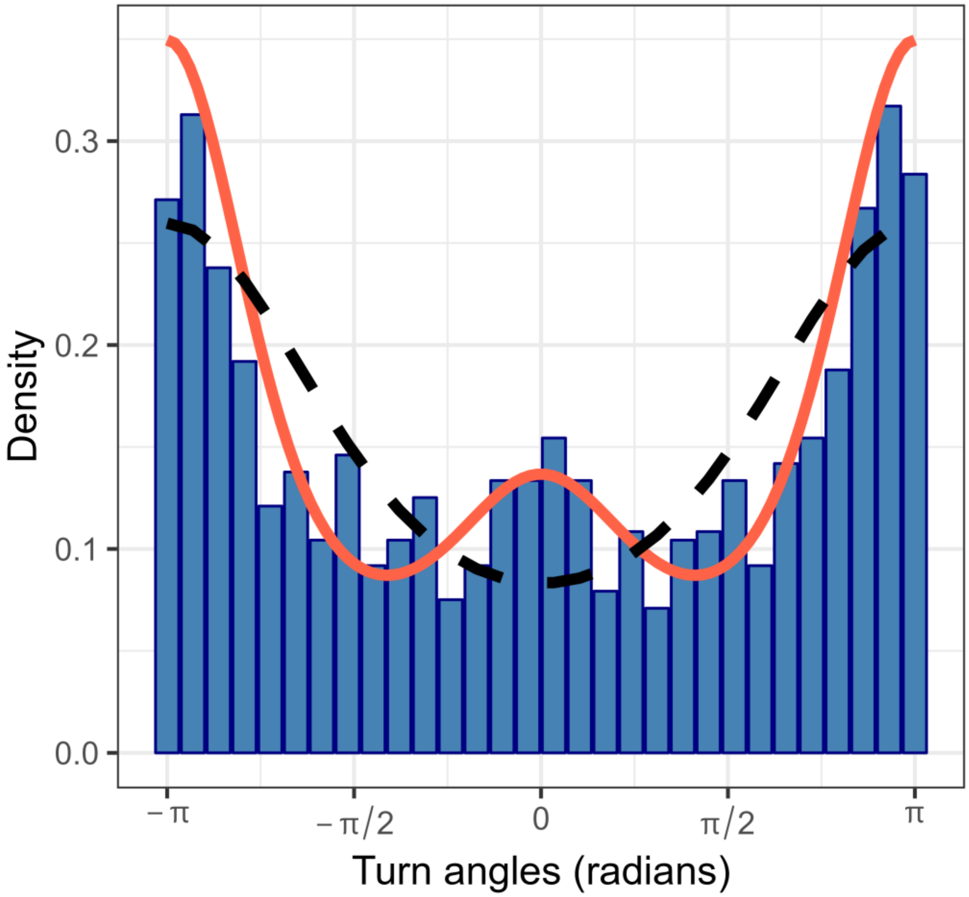

Figure S3. Observed density of turn angles (n = 1,106) for a single caterpillar (blue bars) is fitted nicely by the generalized von Mises distribution (orange solid line), but not by the von Mises distribution (black dashed line). The parameters of the fitted generalized von Mises distribution are κ_1 = 0.469 and κ_2 = 0.428. The parameter of the fitted von Mises distribution is κ = 0.569. The mean of both distributions is centered at π.

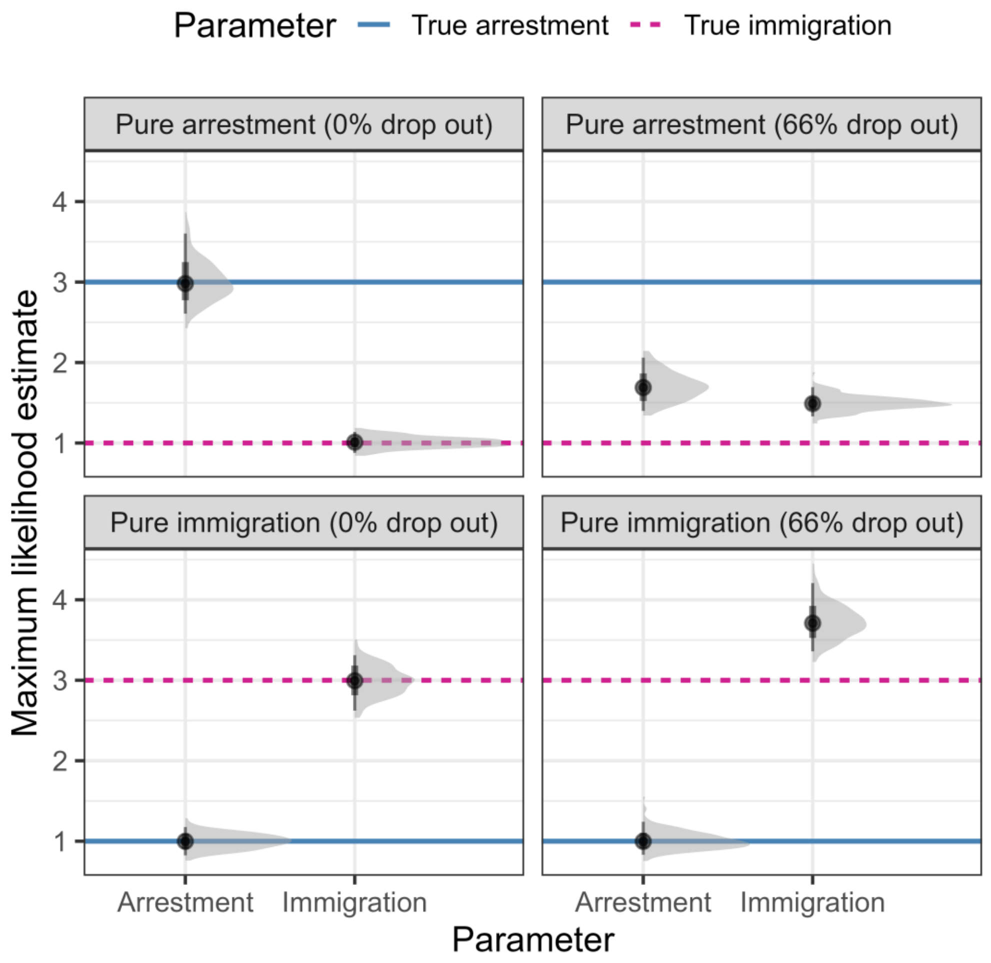

Figure S4. Simulations revealed that integrated step selection analysis can accurately estimate true values of arrestment and immigration when observations are collected at the same frequency at which the simulated processes occur (0% drop out). However, sampling at 1/3rd the frequency (66% drop out), arrestment can masquerade as immigration, but immigration does not masquerade as arrestment. Grey polygons show the distribution of maximum likelihood parameter estimates from models individually fitted to 200 simulated caterpilalr movement tracks. After dropping out, each track constitutes 2,000 observations, simulated under a pure arrestment or pure immigration process (i.e., turn off the other process). Points, thick bars, and thin bars show 50%, 66%, and 95% quantiles.

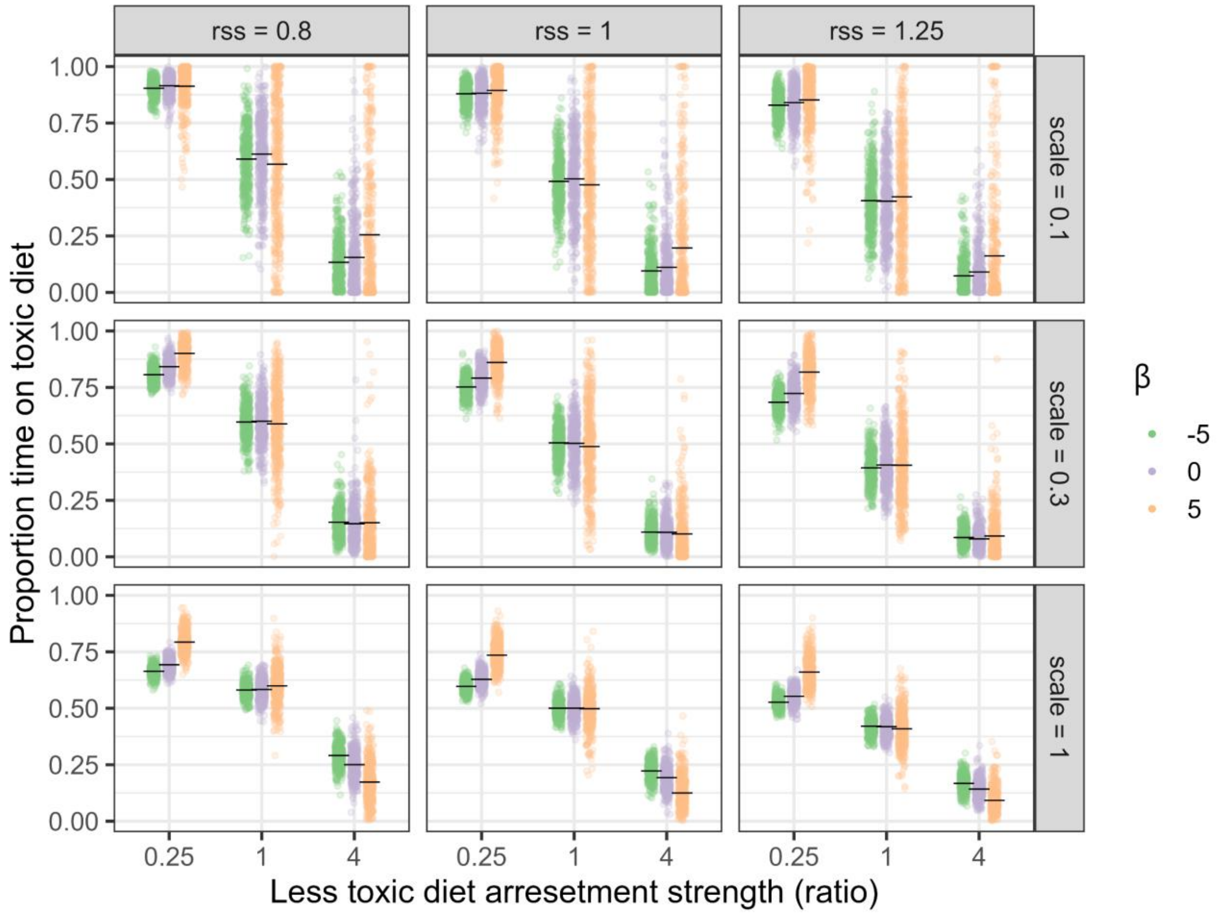

Figure S5. Proportion time on the more toxic diet in an *in silico* experiment with integrated step selection functions parameterized by estimated parameters from observed data. Each point is a unique simulation of 1,000 time steps (~4 days). Black bars represent the group means. 'rss' (relative immigration strength, aka. relative selection strength) denotes the odds ratio of a caterpillar stepping onto a less toxic diet compared to stepping onto a more toxic diet all else being equal.

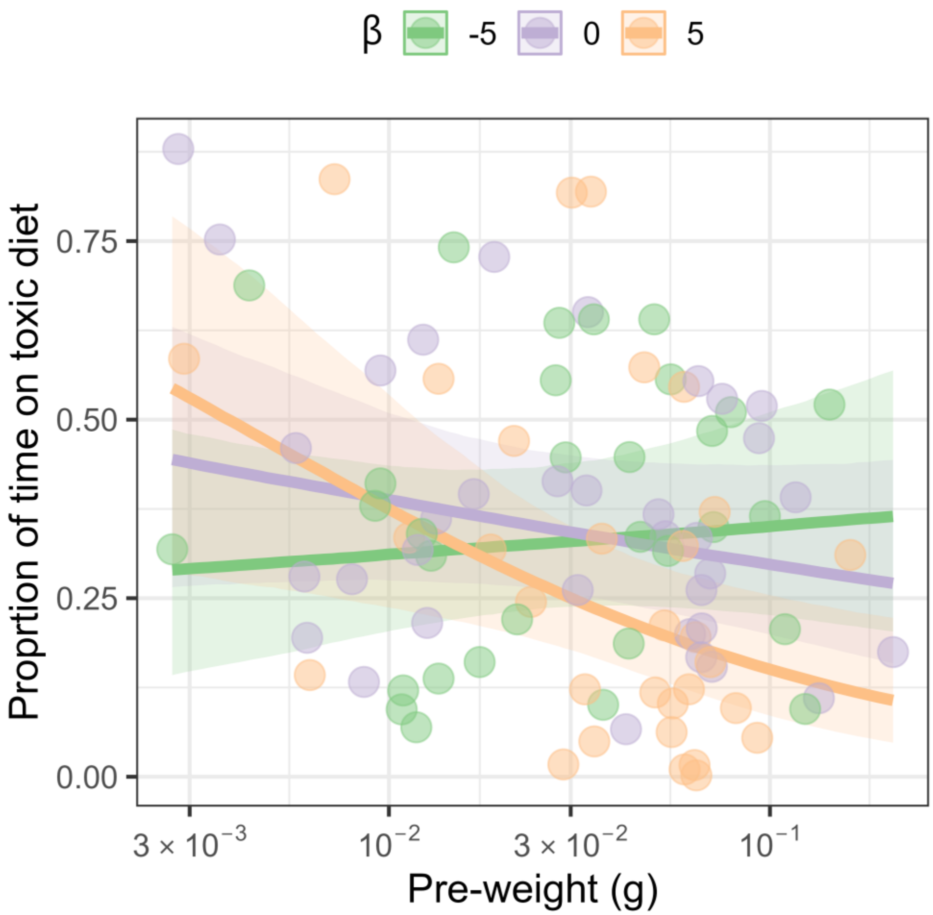

Figure S6. The observed proportion of time spent on the more toxic diet depends on a negative interaction between clusteredness and caterpillar pre-weight. Means and 95% credible intervals of the posteriors are shown as lines and ribbons respectively. Each point is an observed caterpillar (n = 97, marginal R^2 = 0.20).

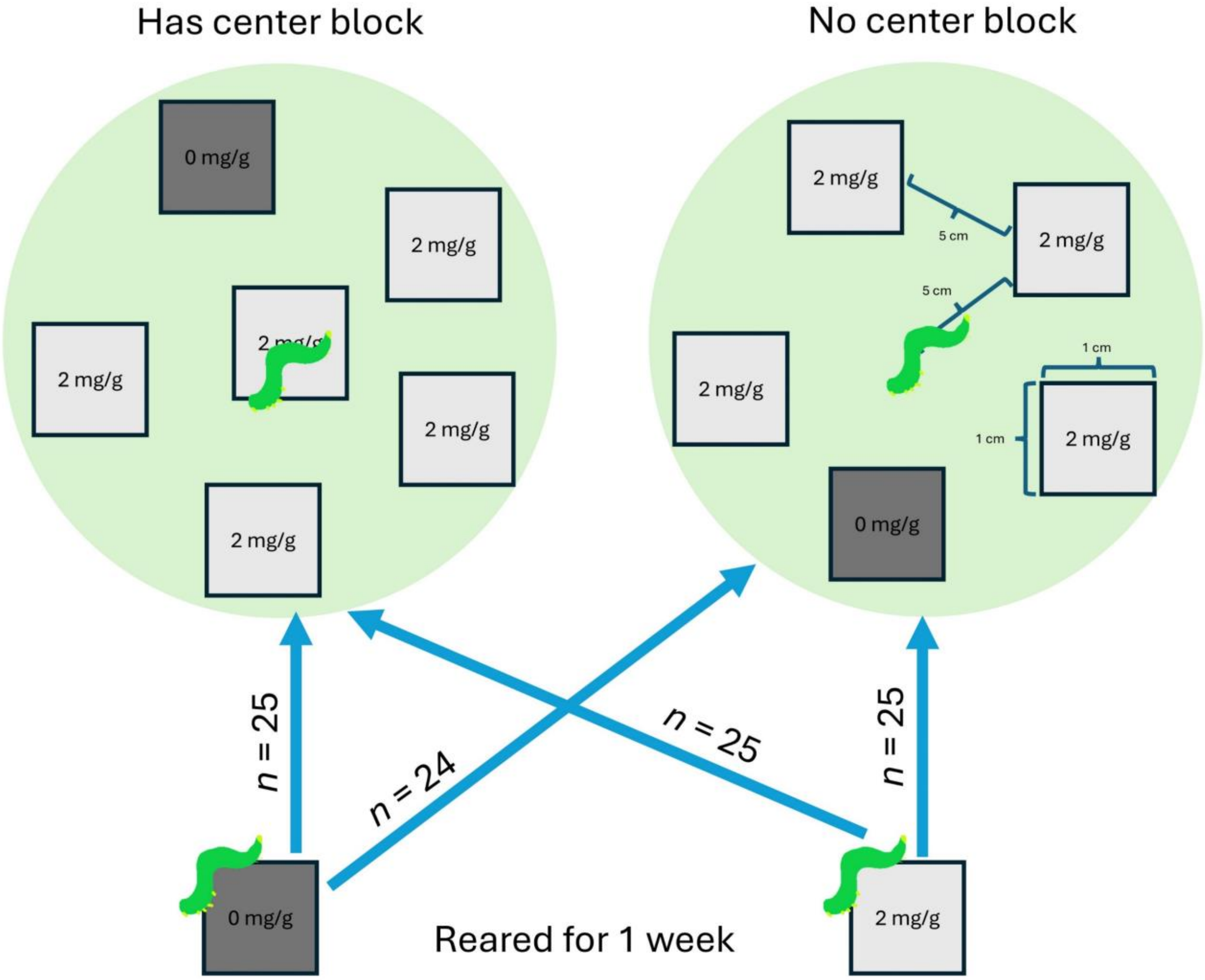

Figure S7. Design of the conditioning experiment. A single unique caterpillar is used in each arena. The doses of xanthotoxin are displayed in the grey boxes. The location of the less toxic diet is randomized. For each caterpillar, we recorded whether it moved to find a new diet and if so, whether the diet was of a high or low xanthotoxin concentration.

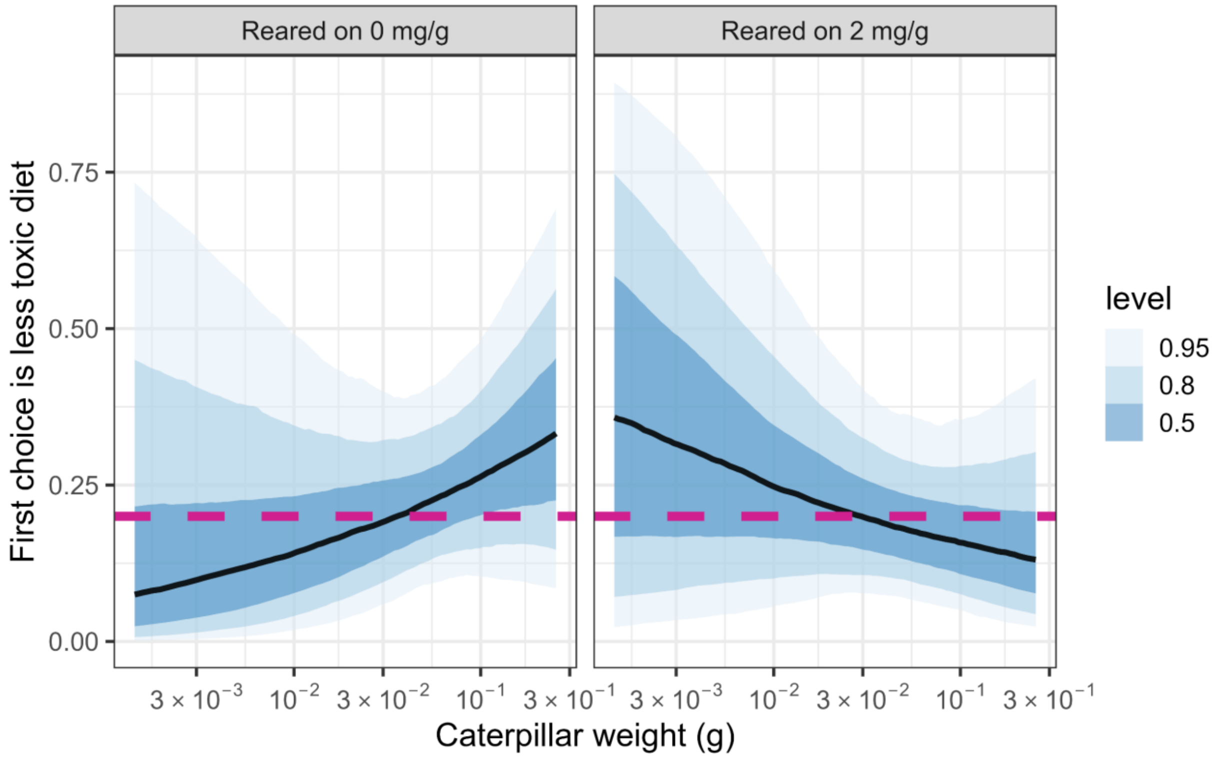

Figure S8. Whether the first choice (excluding no choice) of the caterpillars in the conditioning experiment is the less toxic diet. The lines and ribbons show mean posterior and posterior credible intervals. If the first choice is random with respect to diet toxicity, the caterpillar would move onto the less toxic diet at a rate equal to the environmental availability of the less toxic diet (purple dashed lines at 20%). This is indeed the case for caterpillars of different sizes and caterpillars with no toxin experience (left) and caterpillars with toxin experience (right), indicating that *Trichoplusia ni* likely cannot sense the concentration of xanthotoxin over a long range (~ 5 cm).

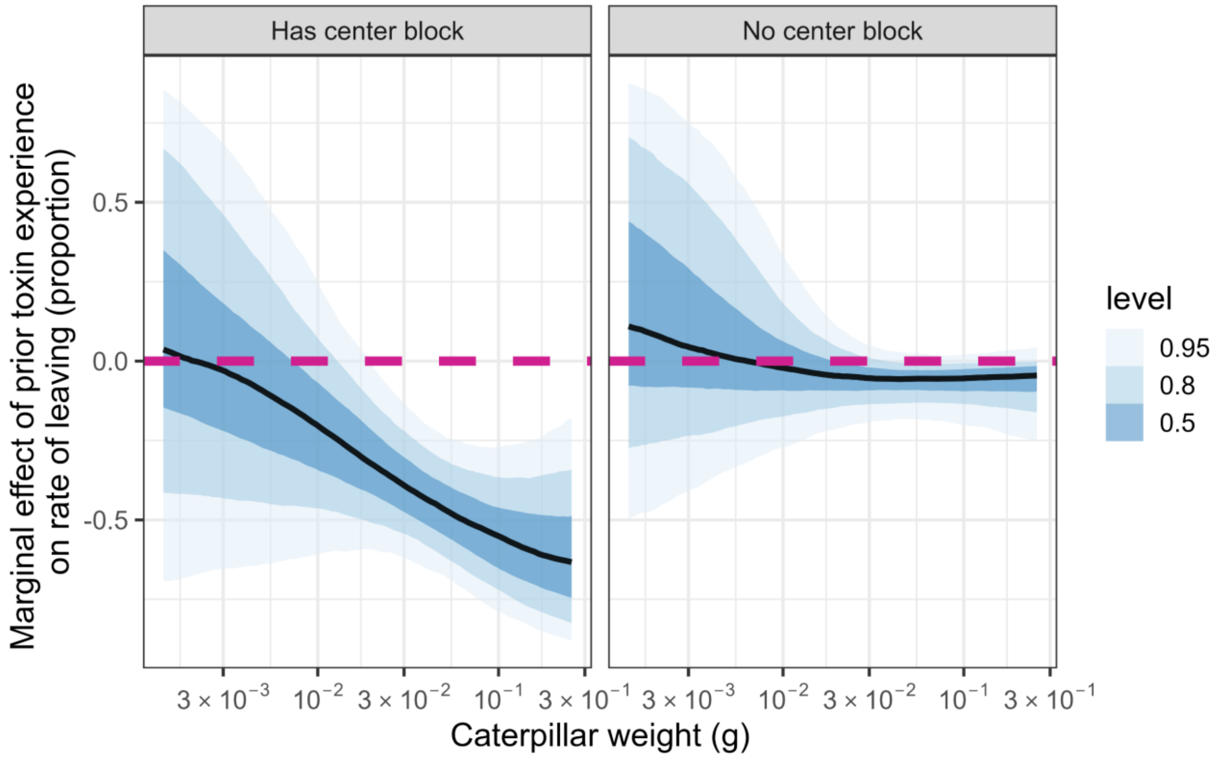

Figure S9. The marginal effect (proportion) of prior toxin exposure on rate of leaving across different caterpillar sizes and center block treatments in the conditioning experiment. The lines and ribbons show mean posterior and posterior credible intervals. Having previous toxin exposure made the caterpillars less likely to move away from a center block that was toxic (no difference is shown as purple dashed lines), especially for larger caterpillars. This result is consistent with the hypothesis that caterpillars use prior experience to evaluate whether the current diet is worth staying on, implying that caterpillars can sense the quality of their current diet (the center block). In the absence of the center block, caterpillars left the center of the ring and found new diets at equal rates between prior experience treatments. This result indicates that caterpillars have equal locomotion abilities, implying that the greater arrestment exhibited by caterpillars with previous toxin exposure when there was a center block was not a result of reduced locomotion abilities.

Video S1. A 30 second video of a single caterpillar's movement and feeding timelapse over 91 hours. The numbers at the bottom right corner display the time in HH:MM. The caterpillar mask predictions are shown in blue. The keypoints for head, middle, and posterior are shown as green, yellow, and red circles respectively. The xanthotoxin concentration treatments are shown as light (1.5 mg/g) or dark squares (0.5 mg/g). The color of the raw timelapse images were transformed to better visualize where the caterpillar has fed.
